# CRISPR Co-Editing Strategy for Scarless Homology-Directed Genome Editing

**DOI:** 10.3390/ijms22073741

**Published:** 2021-04-03

**Authors:** Nina Reuven, Julia Adler, Nadav Myers, Yosef Shaul

**Affiliations:** Department of Molecular Genetics, Weizmann Institute of Science, Rehovot 76100, Israel; julia.adler@weizmann.ac.il (J.A.); nadavmyers@gmail.com (N.M.)

**Keywords:** gene targeting, CRISPR/Cas9, genome editing, endogenous mutagenesis in cell lines, co-editing, scarless selection, temperature-sensitive cell lines

## Abstract

The clustered regularly interspaced short palindromic repeat (CRISPR)/Cas9 has revolutionized genome editing by providing a simple and robust means to cleave specific genomic sequences. However, introducing templated changes at the targeted site usually requires homology-directed repair (HDR), active in only a small subset of cells in culture. To enrich for HDR-dependent edited cells, we employed a co-editing strategy, editing a gene of interest (GOI) concomitantly with rescuing an endogenous pre-made temperature-sensitive (ts) mutation. By using the repair of the ts mutation as a selectable marker, the selection is “scarless” since editing restores the wild-type (wt) sequence. As proof of principle, we used HEK293 and HeLa cells with a ts mutation in the essential *TAF1* gene. CRISPR co-editing of TAF1ts and a GOI resulted in up to 90% of the temperature-resistant cells bearing the desired mutation in the GOI. We used this system to insert large cassettes encoded by plasmid donors and smaller changes encoded by single-stranded oligonucleotide donors (ssODN). Of note, among the genes we edited was the introduction of a T35A mutation in the proteasome subunit PSMB6, which eliminates its caspase-like activity. The edited cells showed a specific reduction in this activity, demonstrating this system’s utility in generating cell lines with biologically relevant mutations in endogenous genes. This approach offers a rapid, efficient, and scarless method for selecting genome-edited cells requiring HDR.

## 1. Introduction

**Figure 1:**
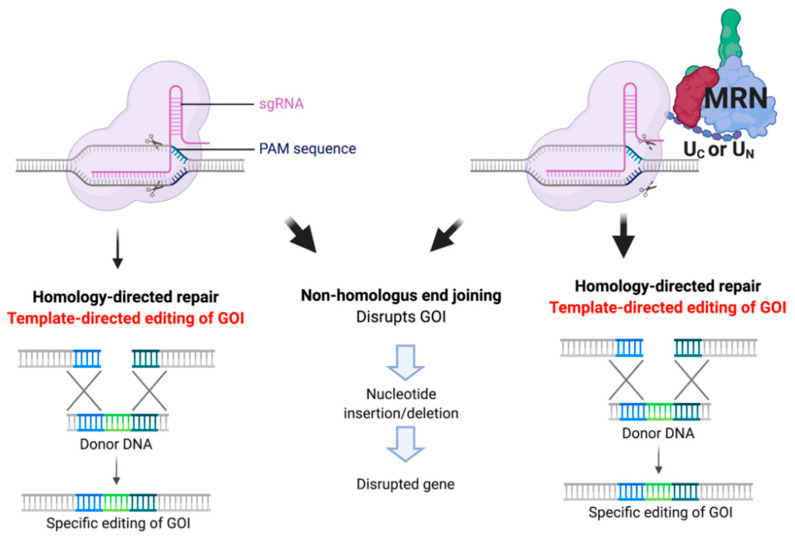
Clustered regularly interspaced short palindromic repeat (CRISPR)/Cas9 gene editing depends on cellular DNA repair pathways. CRISPR/Cas9 creates a double-strand break in the DNA at the specific location dictated by the guide RNA (sgRNA). Cellular DNA repair pathways then repair the break, with nonhomologous end-joining being the predominant pathway in most mammalian cells. Editing a gene according to an exogenously added donor DNA template requires homology-directed repair (HDR), active during the S/G2 cell cycle phase. Fusing a recruitment domain for cellular repair factors, such as the MRN complex, increases HDR-dependent CRISPR editing. Illustrated is Cas9 with a 126aa MRN-recruiting domain from HSV-1 UL12 fused at the N- or C-terminus of Cas9 (U_N_ and U_C_, respectively). Figure created with Biorender.com.

The discovery of the clustered regularly interspaced short palindromic repeat (CRISPR) system and its application in a wide variety of organisms has revolutionized genome editing. Cas, CRISPR-associated (Cas9), is a DNA endonuclease, which cleaves DNA specifically at the target site dictated by its associated guide RNA. Various methods have been developed to simplify the introduction of Cas9 and a programmable guide RNA into the cells, including the modification of the guide so that it is expressed as a single guide RNA (sgRNA), encompassing the sequence specific to the targeted locus (guide) as well as the tracrRNA portion necessary for interaction with the Cas9 protein [1,2,3,4,5]. The CRISPR/Cas9 system provides an elegant and efficient means of creating a specific double-strand break (DSB), which can then be exploited to edit the DNA at the break site. However, repair of the break depends on the endogenous DNA repair machinery. The predominant pathway in most mammalian cells is nonhomologous end-joining (NHEJ), which is highly efficient but error-prone, often making small insertions or deletions (indels) at the repair site that can result in frameshift mutations leading to gene disruption (Figure 1). Thus, the CRISPR system can be used to inactivate (knockout) genes by cutting the DNA and relying on NHEJ to repair it inaccurately. To make specific template-directed edits in the genome, the endogenous homologous recombination (HR) pathway is usually required (Figure 1).

The HR pathway is mostly restricted to the S/G2 cell cycle phase; thus, the window for this type of editing is narrow. Various approaches have been used to boost HDR in mammalian cells in order to improve the yield of HDR edited cells (reviewed in [6,7]). Strategies implemented have included inhibiting NHEJ or inducing HDR genetically and pharmacologically [8,9,10] and by restricting Cas9 cleavage to the S/G2 cell cycle phase by regulating the timing of its degradation or potentially by using an activatable Cas9 [11,12]. Additionally, extra domains have been added to Cas9 that help to promote the HDR process [13,14] or that function as recruiting domains for the cellular HR machinery [15]. Our laboratory has shown that chimeric Cas9 proteins with recruitment domains fused to either the N-(U_N_ Cas9) or C- (U_C_ Cas9) terminus of Cas9 promoted a two-fold increase in HDR-dependent editing (Figure 1) [16]. This short 126aa domain, derived from the HSV-1 UL12 protein, recruits the endogenous MRN complex (Mre11, Rad50, Nbs1).

Since the efficiency of CRISPR editing, even with enhancements, is often low, protocols to select for edited cells have been developed. When using co-editing to insert a selectable marker in addition to editing a gene of interest (GOI), the selected cells will be highly enriched for the second desired mutation as well [17]. Here we show another strategy to select for successfully edited cells, using reversion of an endogenous temperature-sensitive (ts) mutation as the selectable marker. We tested this strategy on a cell line that we had previously developed, with a ts mutation in the essential *TAF1* gene [16]. TAF1 is the largest subunit of the basal transcription complex TFIID (reviewed in [18,19]). It interacts with core promoters and has coactivator, protein kinase, histone acetyltransferase, and ubiquitin-activating/conjugating activities. It also interacts with the other subunits of the TFIID complex. The TAF1 ts HEK293 cell lines that we generated were based on the BHK21 ts13 cell line, having a G690D mutation in TAF1, which leads to cell cycle arrest, and subsequently, cell death, at the restrictive temperature [20]. The HEK293 TAF1 ts cells, bearing the analogous mutation, G716D, were likewise temperature-sensitive and could be repaired with CRISPR editing to restore temperature-resistance [16]. We decided to use these cells for a co-editing strategy, where reversion of the ts gene is the selectable marker. Successful editing of the ts marker gene restores the sequence to wild-type (wt), making this a “scarless” selection. This co-editing strategy was highly efficient in enriching the population for cells successfully edited in the GOI. Furthermore, as the selected cells grew as isolated colonies, it greatly simplified the process of obtaining clones of precisely edited cells.

## 2. Results

### 2.1. Characterization of TAF1 ts Cell Lines

**Figure 2:**
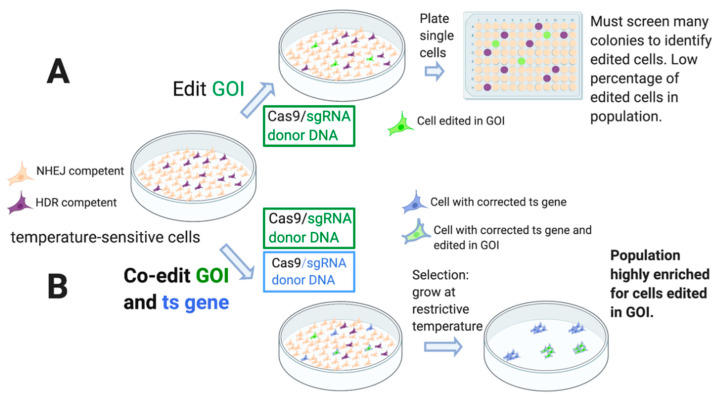
Schematic presentation of co-editing strategy using the repair of a ts gene as a selective marker. (**A**,**B**) Mammalian cells in culture are transfected with editing reagents: Cas9/guide RNA (sgRNA) and donor DNA for editing the gene of interest (GOI). Cas9 and sgRNA can be provided on plasmids or as protein/RNA complexes. Donor DNA can be in the form of plasmids, linear dsDNA, or single-stranded oligonucleotide donor DNA (ssODN). (**A**) Editing cells in a GOI. Specific template-directed editing is inefficient since this type of editing requires homology-directed repair (HDR), and only a minority of cells in culture are HDR-competent. Following transfection of the cells with the CRISPR editing reagents for the GOI, cells are plated as single-cells for analysis of clones. This strategy typically requires the screening of many colonies to identify the desired mutation in the GOI. In this example, 3% of the colonies were edited in the GOI (green colonies). (**B**) Co-editing of ts mutation together with GOI: scarless selection method for productively edited cells. Following transfection of the ts cells with CRISPR reagents for co-editing the GOI together with the ts gene, cells are incubated at 39.5 °C to select for cells that had successfully edited the ts gene, restoring the wt sequence. This selection enriches the population for cells that are active in HDR editing. In this example, 40% of the resulting colonies were also edited in the GOI (green colonies).

We approached this work with two goals in mind: (1) to find a way to select for cells that had undergone genome editing requiring HDR, and (2) to use a selection that would be “scarless”, that is, without noticeable perturbation of the genome, or insertion of foreign genes. To address the first goal, we decided to use a co-editing strategy, editing a gene of interest (GOI) together with an endogenous gene marker that requires HDR for productive editing. To meet the scarless condition, we predesigned a cell line harboring a ts mutation in an essential gene. Editing of the ts gene to restore the wt sequence enables the cells to grow at the restrictive temperature. Since the selectable gene is restored to the wt sequence, the selection is scarless (Figure 2).

As proof-of-principle, we employed HEK293 TAF1ts cells [16] bearing a point mutation (G716D) in the TAFII-250 (*TAF1*) gene on the X chromosome. HEK293 cells have three X chromosomes (https://www.atcc.org/Products/All/CRL-1573.aspx#characteristics; accessed on 1 April 2021) and the TAF1ts clones that we recovered each had one allele with the desired ts mutation, and two alleles with different insertions/deletions (indels), leading to frameshifts (Supplementary Figures S1 and S2). *TAF1* also has an intronless paralog in the hominoid lineage, *TAF1L*, on chromosome 9, likely generated via a duplicative retrotransposition event [21]. TAF1L protein expression is restricted to the testis and to bipolar neurons [21,22]. Furthermore, most commonly used cell lines express no or barely detectable levels of TAF1L. The Protein Atlas (http://www.proteinatlas.org; accessed on 1 April 2021) shows expression levels of 0.1 for TAF1L in HEK293 and HeLa, compared to 13.9 and 13.1, respectively, for TAF1 [22]. Since the region targeted by the guide RNA to create the ts mutation is identical in both *TAF1* and *TAF1L*, we also analyzed *TAF1L* for mutations resulting from cutting with the Cas9/sgRNA. These analyses showed that, like *TAF1*, *TAF1L* had been edited in the TAF1 ts clones (Supplementary Figure S3). In HEK293 TAF1ts clone 1, *TAF1L* had also been edited to create a ts allele. In contrast, in HEK293 TAF1ts clone 11, the *TAF1L* alleles had indels that would eliminate expression of a functional protein and would also prevent further targeting by the sgRNA specific to the wt or the ts TAF1. In this clone, the clear identification of the unique edited alleles of *TAF1* and *TAF1L* suggested that the chromosomes 9 and X that were targeted appeared to have remained intact, except for the small indels and specific ts mutation.

We next analyzed the *TAF1* and *TAF1L* loci in the ts cells following CRISPR/Cas9-mediated repair of the ts mutation and selection of cells at 39.5 °C. We isolated ts-rescued clones and sequenced the targeted TAF1ts locus and TAF1L (Supplementary Figures S4 and S5). The TAF1 ts allele was restored to the wt sequence in the rescued clones (Supplementary Figure S4). In the rescued HEK293 TAF1 ts clone 11, the alleles of both *TAF1* and *TAF1L* that had indels from the original CRISPR targeting were unchanged. Thus, CRISPR-mediated editing of the ts mutation in these cells was restricted to the TAF1 G716D ts allele.

The ts cells growing at 37 °C were morphologically similar to the naïve HEK293 cells and the rescued cells; however, while the naïve HEK293 and rescued clones grew well at the restrictive temperature of 39.5 °C, the ts cells died after 5 days of incubation at 39.5 °C (Supplementary Figure S6) and had detached from the plate after 7 days of incubation (Supplementary Figure S7A). Although freshly plated ts cells had a longer lag time than naïve or rescued cells before commencing exponential growth at 37 °C (Supplementary Figure S7B), the exponentially growing ts cells doubled at essentially the same rate as naïve HEK293 cells (32 h for ts clones 1 and 11, and 34.5 h for naïve HEK293) (Supplementary Figure S8).

Having shown that we could successfully introduce the TAF1 ts mutation and repair it using HEK293 cells, we next wanted to know if the same method could be used in other cell lines. For this purpose, we engineered the same mutation in *TAF1* in HeLa cells and sequenced the *TAF1* and *TAF1L* loci (Supplementary Figure S9). HeLa cells have diploid X with additional rearrangements [23]. The HeLa TAF1 ts clone that we isolated had one TAF1 ts allele and a deletion leading to functional knockout in the other (Supplementary Figure S9). The HeLa ts cells grew at 34 °C and at 37 °C, but like the HEK293 TAF1ts cells, died when incubated at 39.5 °C (Supplementary Figure S10A). CRISPR-mediated rescue of the ts allele yielded colonies growing at 39.5 °C, with no spontaneous revertants observed (Supplementary Figure S10B).

### 2.2. Co-Editing of the TAF1 ts Cells

**Figure 3:**
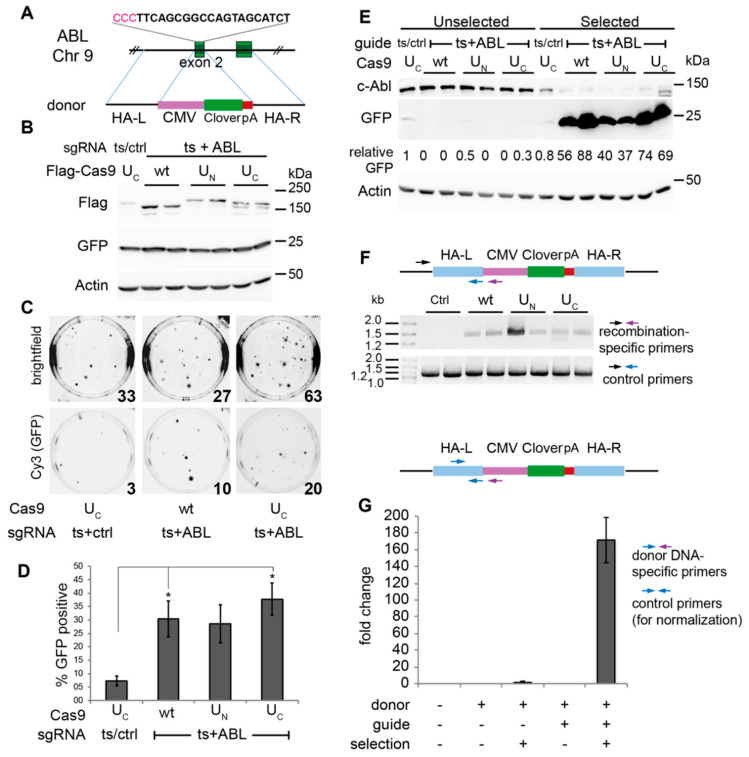
Co-editing of ts gene and *ABL1* locus improves recovery of ABL-edited cells. (**A**) Schematic of the construct targeting *ABL1* to insert a 1.95 kb CMV-CloverGFP-pA cassette into exon 2. The guide sequence is shown above in bold, with the PAM sequence in pink (the guide is on the minus strand). The donor plasmid has the cassette flanked by 1 kb homology arms. (**B**–**F**) Co-editing of TAFts and CMV-CloverGFP cassette in ABL1. HEK293 TAF1 ts (clone 1) cells were transfected in biological duplicates with CMV-Clover in ABL donor plasmid, ssODN for the rescue of TAF1ts, and plasmids for expression of Flag-Cas9 constructs and sgRNAs targeting TAF1ts and ABL1, or TAF1ts and a non-targeting sgRNA for the control. Two days post-transfection, cells from each well were replated into two dishes, with one maintained at 37 °C and passaged every 4–5 days over the course of the experiment (one month), with samples taken for SDS-PAGE and immunoblot at the first passage (shown in panel **B**). The other dish was transferred to 39.5 °C. The medium was changed as needed, but these cells were not passaged. (**C**) GFP expression in heat-selected colonies was assessed by photographing the cells using the Cy3 filter (GFP) and brightfield. The numbers of colonies are indicated at the lower righthand corners of the images. (**D**) Summary of GFP-expression in heat-selected colonies, *n* = 2, error bars represent SEM, * *p* < 0.05. (**E**) Pools of the colonies that had grown at 39.5 °C (“selected”) and samples of the cells continuously grown at 37 °C (“unselected”) were analyzed by SDS-PAGE and immunoblot. (**F**) Analysis of genomic DNA of heat-selected cells. Genomic DNA from the pools of selected cells was PCR-amplified using the control or recombination-specific primer pairs indicated. The recombination-specific primer set generates a 1.4 kb band, and the control set a 1.1 kb band. (**G**) qPCR was performed on genomic DNA from the pools of selected and non-selected cells using primers specific for the left homology arm and the CMV sequence within the donor plasmid. Primers specific to genomic DNA were used for normalization. *N* = 2, error bars represent SEM.

To test the co-editing protocol, we edited endogenous genes, using either plasmid donor DNA for large insertions or single-stranded oligonucleotide donors (ssODN) for small insertions or point mutations. The first gene targeted was *ABL1*, and we inserted a CMV-Clover (GFP) cassette into the second exon of the gene. This 1.9 kb cassette has its own promoter (CMV) and poly-A signal for GFP expression. Insertion of the cassette should disrupt the transcription of the *ABL1* gene itself (Figure 3A). We transfected the cells with the donor DNA for modifying *ABL1*, the ssODN for TAF1ts rescue, together with plasmids expressing Cas9 and sgRNA.

In addition to a “wild-type” Flag-tagged Cas9, we also used two other Cas9 constructs that enhance HDR by recruiting the endogenous MRN complex (Figure 1). The recruiting domain was fused at either the N-terminus (U_N_) or C-terminus (U_C_) of Cas9. We wanted to know whether these constructs would further improve the yields of correctly edited cells. Western blot analysis of samples taken early after transfection showed equal levels of transfected Cas9 constructs and expression of GFP from the cassette on the donor plasmid (Figure 3B). At this point, the transfected cells were split into two plates, with one incubated at 39.5 °C to select for cells that had successfully edited the ts gene, and the other maintained at 37 °C. Cells that had rescued the TAF1 ts gene formed colonies at 39.5 °C and 24–44% of these colonies expressed GFP (Figure 3C,D). While U_C,_ the MRN-recruiting Cas9 construct, led to twice as many heat-resistant colonies compared to the wild-type Cas9, the percentage of co-editing was similar among all the Cas9 constructs. To determine whether the co-editing protocol succeeded in enriching for successfully edited cells, we used Western blotting to compare the GFP expression in pools of the selected cells, compared with the unselected cells that had been maintained at 37 °C. The analysis showed that the background GFP expression from the donor plasmid in the control transfection was low (Figure 3E), showing that after passaging the cells for a month, the cells had mostly lost the transiently transfected donor plasmid. However, GFP expression from unselected co-edited cells was also mostly undetectable, indicating a very low efficiency of editing. FACS analysis of these cells indicated that <0.5% of the unselected edited cells expressed GFP. In contrast, cells in the selected population were highly enriched for GFP expression, consistent with the GFP-expression analysis of the colonies. Quantification of GFP expression in the Western blot showed an approximately 50–150-fold increase in GFP in the selected, co-edited cells. In addition, knockout of c-Abl was evident in this population (Figure 3E). To verify that the GFP cassette was inserted at the correct location, we performed PCR on the genomic DNA of the pooled selected colonies (Figure 3F). This analysis showed that the cassette was inserted faithfully in the pools of cells selected at high temperature and was not detected in the control cells, where the sgRNA for *ABL1* was not included. To further quantify the enrichment of edited cells in the selected pool, we performed qPCR on total DNA samples (includes genomic DNA and free plasmid DNA in the cells) using primers that detect the donor DNA (Figure 3G). While residual donor DNA gave a small signal in the control selected cells, the signal was 73-fold higher in the co-edited, selected cells. We could not compare the unselected co-edited cells to the selected ones since the unselected cells gave no signal in this assay. Using the most conservative assessment, if we assume that the efficiency of editing was approximately 0.5% in the unselected, co-edited cells, expression of GFP in 22–44% of the selected cells represents a 70-fold enrichment by the co-editing strategy.

**Figure 4:**
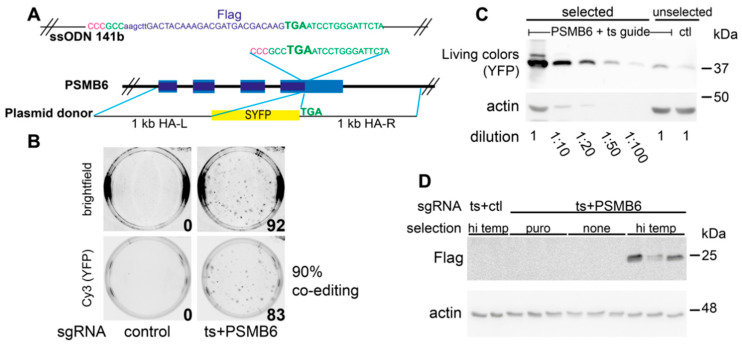
Co-editing of *PSMB6* gene in HEK293: insertion of large and small tags. (**A**) Schematic representation of the targeted *PSMB6* locus with a donor plasmid for YFP insertion and ssODN for Flag tag insertion. The stop codon of PSMB6 is indicated in bold. The guide sequence is in green, with the PAM sequence in pink (note that the guide is the minus strand). The region of the plasmid donor DNA with the 1 kb left and right homology arms (HA) and SYFP insert are shown. (**B**,**C**) Co-editing of HEK293 TAF1ts to revert ts mutation and insert YFP at PSMB6 C-terminus. HEK293 TAF1ts cells (clone 11) were transfected with donor DNA for ts correction and PSMB6-YFP, and Cas9/guide plasmids targeting TAF1ts and PSMB6 (co-edited), or nonspecific sgRNAs (control). Transfected cells were replated into 6 cm dishes and incubated at 39.5 °C. Colonies were photographed 20 days later using the Image Quant LAS 4000 system, using both the Cy3 filter to view YFP fluorescence and brightfield. (**C**) Cells were plated at 37 °C (unselected) or at 39.5 °C (selected), and pools of cells were analyzed by Western blotting. Dilutions of the selected cell extract were compared to the controls, showing a 50-fold enrichment of PSMB6-YFP co-editing.PSMB6-YFP is a 50 kDa protein but migrates at 37 kDa when samples are not boiled prior to loading on SDS–PAGE. This improves the detection of YFP by the Living Colors antibody. (**D**) Co-editing of ts and PSMB6-Flag in HEK293 TAF1ts. HEK293 TAF1 ts (clone 11) were transfected in triplicates with Cas9/guide plasmids indicated, ssODN for TAF1 and for PSMB6-Flag, and pEFIRES, a plasmid providing puromycin resistance. Two days post-transfection indicated cells were treated with 0.5 μg/mL puromycin overnight to select for transfected cells. The following day, cells were replated into 6 cm dishes and incubated at 37 °C (puro and no selection control) or at 39.5 °C. Cells were analyzed by SDS–PAGE and immunoblotting with the indicated antibodies.

To further validate the co-editing protocol, we modified the endogenous *PSMB6* gene, which encodes a subunit of the core 20S proteasome, to express PSMB6 fused at its C-terminus to YFP. We had previously designed the plasmid donor and sgRNA for this system, [16] (Figure 4A), and demonstrated that the endogenously produced PSMB6-YFP was incorporated into functional proteasomes. As in Figure 3, we compared co-edited cells incubated at the restrictive temperature (39.5 °C) to the unselected cells grown at 37 °C. YFP was expressed in 70–90% of the heat-selected colonies (Figure 4B, Supplementary Figure S11). The percentage of co-editing was similar for the U_N_ and U_C_ constructs (Supplementary Figure S11). To verify that the YFP expression was indeed from the PSMB6-YFP, the colonies were harvested as a pool and were analyzed by Western blotting (Figure 4C) in comparison to pools of unselected cells. The results show that the selected cells were greatly enriched for PSMB6-YFP. By loading dilutions of the selected cells’ extract, it could be estimated that the enrichment achieved was in the range of 50-fold (Figure 4C).

We next tested co-editing, where the template for the GOI is a ssODN. To this end, we again edited the C-terminus of PSMB6, adding a Flag tag (Figure 4A). In this setting, the addition of the Flag tag by western blotting was undetectable in the co-edited cells that were not selected at high temperature, including those that had been enriched for successful transfection (Figure 4D). However, the 39.5 °C-selected pools of cells expressed the Flag-tagged PSMB6, and analysis of the genomic DNA showed the edited allele in 5–6% of the reads (Supplementary Figure S12). Assuming that most of the editing was likely to be mono-allelic, this analysis indicated that 5–12% of the cells expressed PSMB6-Flag.

**Figure 5:**
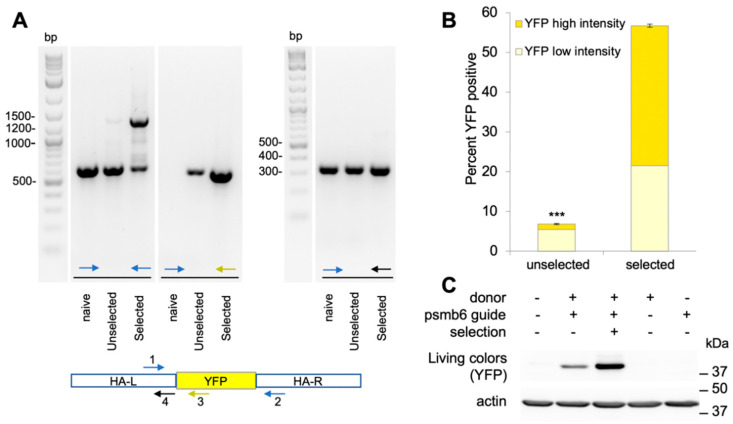
Editing of PSMB6-YFP is improved in HeLa TAFts cells. HeLa TAFts cells were transfected with Cas9/sgRNA encoding plasmids and with donor DNA. Two days after transfection, cells were placed at restrictive or permissive temperatures. (**A**) PCR analysis of HeLa PSMB6-YFP cells. Genomic DNA from naïve HeLa cells and co-edited non-selected or selected pools were subjected to PCR using the primers indicated. The “1” forward and “2” reverse primers generate a 600 bp fragment using naïve genomic template and a 1500 bp fragment with the YFP insertion. Using the YFP reverse primer (“3”) and “1” forward primer, the expected fragment is 1050 bp. Using “1” forward and “4” reverse primers, a 300 bp fragment is generated and used for normalization. (**B**) FACS analysis of HeLa PSMB6-YFP co-edited cells. Cells were analyzed by FACS with 30,000 cells per point, *n* = 3 *** *p* < 0.0001. (**C**) PSMB6-YFP is enriched in HeLa co-edited cells. Pools of selected (39.5 °C) and unselected (37 °C) cells transfected with the reagents indicated were analyzed by SDS–PAGE and immunoblotting with the indicated antibodies.

We next tested our ts co-editing protocol on the HeLa TAF1ts cells, using the PSMB6-YFP model (Supplementary Figure S13A). As we found with the HEK293 TAF1 ts cells, co-editing with the selection at 39.5 °C greatly enriched the population for cells that had accurately edited the cells. PCR analysis of the genomic DNA showed that the selected cell pool was enriched for the PSMB6-YFP insertion (Figure 5A). This result was confirmed by FACS analysis (Figure 5B) and by Western blotting (Figure 5C). The FACS analysis showed that 7% of the unselected population compared to 57% of the selected cells expressed PSMB6-YFP, an 8-fold enrichment. Furthermore, the FACS analysis showed two peaks of YFP (Figure 5B and Supplementary Figure S13B), with one having an average intensity that was double that of the other, suggesting that the higher intensity could indicate biallelic insertion. Of the YFP-positive cells, the unselected population had only 20% of the cells with high-intensity YFP, whereas in the selected population, the high-intensity cells were 62% of this population. These data show that co-editing can provide efficient gene editing in HeLa TAF1ts.

**Figure 6:**
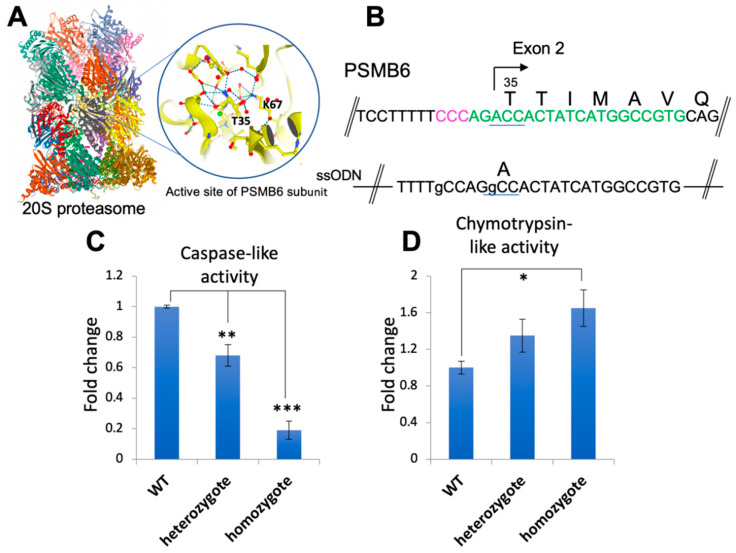
Co-editing generates PSMB6 T35A mutant cells with reduced proteasome caspase-like activity. (**A**,**B**) Threonine 35 at the active site of the proteasome PSMB6 subunit is mutated to alanine using sgRNA (marked in green) and ssODN shown. (**C**,**D**). Caspase-like and chymotrypsin-like activities of control HEK293 TAF1ts repaired cells, and the T35A PSMB6 mutants were measured using the fluorescent substrates Z-LLE-AMC and Suc-LLVY-AMC as described in Methods. *N* = 4, * *p* < 0.05, ** *p* < 0.01,*** *p* < 0.001. The structures for the 20S proteasome and the PSMB6 subunit were derived from RSCB PDB ID 5LE5 [26,27].

We next challenged the system by engineering a point mutation in an endogenous gene, again targeting the human *PSMB6*. This subunit possesses caspase-like (CL) activity, and mutation of the active site threonine to alanine eliminates this activity [24,25] (Figure 6A,B). Of 26 co-edited clones tested, 12 (46%) had one T35A allele. No clones had biallelic T35A mutations, but 11/12 were wild-type for the remaining allele, and 1/12 had a frame-shifting indel in the second allele. We tested the CL activity of proteasomes from wt ts-rescued cells, from a heterozygote (one wt, one T35A allele), and from the clone that had one T35A allele and one knocked-out allele (“homozygous” for the T35A mutation). CL activity was reduced proportionally according to the genotype, with the heterozygote displaying an intermediate activity and the “homozygote” having basal activity (Figure 6C). The loss of CL activity was specific since no loss of chymotrypsin-like activity was seen in the mutants (Figure 6D). All three cell lines displayed similar morphology (Supplementary Figure S14). These data demonstrate that the ts co-editing system can produce biologically relevant mutations in endogenous genes. In conclusion, co-editing of TAF1 ts cells is a robust and scarless method to enrich for CRISPR-edited cells that depend on HDR for precise editing.

## 3. Discussion

We have developed a new strategy for rapid and efficient HDR editing. This system offers several advantages over current technologies. First, the selection is based on reversion of a temperature-sensitive gene, and thus once it is repaired, the result is “scarless,” and the edited cells are modified only in the GOI. Second, the reagents needed for editing the ts mutation, namely Cas9, sgRNA, and ssODN, can be delivered without the use of plasmids, as the Cas9/sgRNA can be delivered as a ribonucleoprotein complex. This is beneficial for systems where it is imperative to avoid the risk of random integrations of foreign DNA, which can occur with plasmids or double-stranded DNA fragments. Third, the method does not require expensive reagents. Although this method requires first engineering a ts mutation in a cell line of interest, once such a cell line is produced, it can be used to make HDR-dependent mutations encoded by ssODNs or plasmid-based donors quickly and efficiently. The HEK293 and HeLa TAF1ts cells are useful reagents and proof-of-principle that such a strategy may be used in other cell lines and with other ts genes. Fourth, the colonies that arise after selection at high temperatures are clones, and therefore, laborious single-cell cloning is avoided.

Precise genome editing can be very inefficient, and strategies have been developed to improve the yield and accuracy of correctly edited cells. These strategies fall into two basic categories: those geared to improve the editing process itself and those aiming to select for successfully edited cells. In order to improve the editing process itself, methods that shift the balance between HDR and NHEJ have been used (reviewed in [6,7]. These include inhibiting NHEJ or promoting HDR genetically or pharmacologically. Since HDR is active in the S-G2 phases of the cell cycle, another way to shift the balance between HDR and NHEJ is to restrict Cas9 expression or activity to these phases of the cell cycle by, for example, fusing Cas9 to a fragment of the cell cycle-regulated protein geminin [11]. Other approaches have addressed the problem of NHEJ by avoiding the double-strand break altogether and using a Cas9 partially or completely devoid of its endonuclease activities. Cas9 fused to base editors has been shown to specifically edit targeted locations [28]. Furthermore, pairing a nickase Cas9 with reverse transcriptase and an RNA-encoded template has served to accurately edit targeted regions according to the associated template [29]. Other tactics have used active Cas9 fused to protein domains that can locally modulate the DNA damage response at the break site. Examples of this approach include inhibiting NHEJ by fusing Cas9 to a dominant-negative 53BP1 [14], to factors involved in HDR [13] or capable of recruiting these factors [15,16]. Since the targeted cells and genomic loci each have their own unique characteristics, each of these systems may provide the best solution in a particular cell line, in vivo system, or specific gene edit, but not necessarily in others. Therefore, which system to choose would depend on the particular demands and characteristics of an editing project, some of which can only be determined empirically. CRISPR editing of human cells for therapeutic purposes, particularly if the cells are the patient’s autologous cells, must be rapid, accurate, and with minimal exposure to foreign DNA. A recent study compared strategies for improving HDR when correcting the mutation causing sickle cell anemia in human hematopoietic stem and progenitor cells (HSPCs) [30], finding that Cas9 fusions with a geminin fragment yielded the best HDR/NHEJ ratio, although a Cas9 construct with both the geminin and UL12 MRN-recruiting domains gave the highest percentages of HDR.

When using a selection strategy to isolate productively edited cells, the best method will also depend on the particular constraints of the system used. There have been several different strategies published that use co-editing of a selectable marker together with a gene of interest. Some have integrated a foreign gene, such as a fluorescent protein or antibiotic-resistance marker [17]. In other cases, an endogenous gene has been modified in order to make the cells resistant to a drug or medium condition, such as modification of the Na^+^/K^+^ ATPase for resistance to ouabain [31] and editing of the diphtheria toxin receptor to enable selection with diphtheria toxin [32]. The advantage to these approaches is that they can be used in naïve cells, including, potentially, primary human cells for gene therapy. The disadvantage is that the mutations either add foreign genes or mutate endogenous proteins, and thus they are not “scarless”. Whether these mutations will impinge on the activity or viability of these cells will need to be checked for the specific conditions and demands of the assay or application. Another approach used repair of a puromycin-resistance gene encoded by a plasmid as a selectable marker [33]. The advantage of this system is that the selectable gene is expressed transiently on a plasmid. However, as with all editing systems involving plasmids, it will be important to verify that the plasmid itself has not been integrated into the genome.

Our approach, using a temperature-sensitive gene as the selectable marker, avoids some of the issues encountered with the systems mentioned above but also has limitations of its own. An advantage is that large plasmid-encoded inserts can be edited into the genome accurately, something that is not possible with strategies such as base editing [28] or prime editing [29]. However, when using our strategy, as with any co-editing strategy based on double-strand breaks, it is important to verify that the clones produced do not have any unwanted mutations stemming from off-target cutting by the Cas9/sgRNAs, and that no translocations have occurred between the edited chromosomes. An obvious limitation of our system is that the desired mutation in the GOI cannot itself cause temperature-sensitivity. However, the main limitation in our system is that the selectable ts mutation must first be introduced into the desired cell line. Yet, while difficult, engineering ts laboratory cell lines, or potentially, clinically relevant cell lines, can simplify the later production of many HDR-dependent edits in these cells. In this study, we used the TAF1 ts mutation, but ts mutations in other essential genes could also be employed. Our proof-of-principle using HEK293 and HeLa cells have already provided useful reagent cell lines for making mutations in these backgrounds. The advantage to the ts approach is that it is scarless in that the endogenous selectable gene is restored to wild-type upon selection. The caveat here is that in the HEK293 and HeLa cell lines that we have made, the X chromosome is polyploid, and the ts cell lines we isolated each had one ts allele and indels leading to *TAF1* gene knockout in the remaining alleles. Additionally, the *TAF1L* gene was also edited. However, since the knockout alleles are constant in the ts rescued co-edited cells, the cell lines generated using this system have the same *TAF1* genotypes. Since there is only one allele that can be edited to restore growth at high temperatures, this precludes the possibility of getting unexpected TAF1 products in co-edited cells.

Another potential concern for strategies such as this one, is that the “knockout” alleles may produce a truncated protein that could interfere with the activity of the wt protein. In order to verify that this was not happening in our system, we amplified the targeted region of TAF1/TAF1L from cDNA of the rescued clone of HEK293 TAF1ts clone 11. Sequencing of this sample showed that only wt mRNA was present, indicating that no truncated proteins should be produced from the non-functional alleles (Supplementary Figure S15).

A notable advantage of the TAF1 ts co-editing system is that the selection is very stringent; we have not observed a random escape of the ts mutation. Furthermore, repair of the cells depended on the ssODN and the sgRNA, meaning that the selection is for cells capable of editing an endogenous gene in a template-directed manner. For some co-editing strategies [34], it is enough to knock out the selective gene to survive the selection, meaning that the selection is not specific for template-directed repair.

In each of the systems we tested, we saw a percent of co-editing that was characteristic of the targeted gene of interest and the type of edit. For example, when targeting the *ABL1* gene with the inserted CMV-Clover cassette (Figure 3), an average of 35% of the co-edited colonies were positive for Clover GFP. In contrast, the percentage of co-editing was much higher with creation of PSMB6-YFP, where an average of 75% of the colonies expressed PSMB6-YFP (Figure 4 and Supplementary Figure S11), while using the same guide but a different donor (ssODN for Flag tag) gave much lower editing. Thus, the percent efficiency of obtaining the desired HDR-dependent mutation appeared to depend on the particular features of the target gene and the editing reagents. The data from the modified Cas9 constructs further supports this conclusion. The modified Cas9 constructs U_N_ and U_C_ have a short 126aa domain capable of recruiting the endogenous MRN complex [16]. The MRN complex is necessary for HDR, and thus by recruiting it directly to the site of the Cas9-induced DSB, HDR-dependent editing is increased two-fold. In the present study, the MRN-recruiting Cas9 constructs U_N_ and U_C_ typically resulted in two-fold more colonies than what we obtained with wild-type Cas9, consistent with what we had seen previously [16]. However, the percentage of successfully co-edited target genes stayed consistent and characteristic of the target gene. The improvement in HDR using the MRN-recruiting constructs did not change the ratio of repair between the selection gene and target gene. This suggests that the rate of success in co-editing a GOI has to do with the relative efficiency of the sgRNAs/template for the ts gene and the GOI. Consistent with this, a co-editing system based on HDR-dependent repair of a plasmid-based reporter (HDR-USR) [33] showed improved yields with more efficient sgRNAs for the plasmid reporter.

A further advantage of the ts co-editing system is that the temperature-resistant edited cells grow as colonies that arose from single-cells. This means that no further single-cell cloning is required to isolate clones. Using this system, verified mutant cell lines can be obtained in as little as three weeks. Thus, the TAF1ts co-editing system offers a simple, robust, efficient and essentially scarless means to obtain HDR-dependent editing in mammalian cells.

## 4. Materials and Methods

### 4.1. Cells and Cell Culture

Human embryonic kidney cells HEK293 (ATCC^®^ CRL1573™), HeLa (ATCC^®^ CCL-2) and HEK293 TAF1ts [16] cells were grown at 37 °C in a humidified incubator with 5.6% CO2 in Dulbecco’s modified Eagle’s medium (DMEM; GIBCO, Life Technologies, Thermo Scientific, Waltham, MA, USA) supplemented with 8% fetal bovine serum (GIBCO), 100 units/mL penicillin, and 100 μg/mL streptomycin. The restrictive temperature used for the TAF1ts cells was 39.5 °C. Puromycin was from GoldBio (St. Louis, MO, USA). Light microscopy photographs of cells were performed using an Olympus (Tokyo, Japan) IX70 microscope connected to a DVC camera. Cell colonies expressing YFP or Clover GFP were visualized using the ImageQuant LAS 4000 (GE Healthcare, Piscataway, NJ, USA). The Incucyte^®^ SX1 live-cell analysis system (Sartorius) was used to photograph cells and quantify cell proliferation and viability.

### 4.2. Calculation of Cell Doubling Time

Cells were plated in 24 well plates at 14,000 cells/well in quadruplicate. Cells were photographed in situ by the Incucyte system, at 10 ×, with 25 images per well, every 2 h. The Incucyte analysis software calculated percent confluence. Log2 of the confluence was plotted vs. time for cells in exponential growth, with the slope of the line being the doubling time of the cells.

### 4.3. Plasmids and Transfection

The SpCas9/sgRNA expression plasmids were described in [16] (Addgene plasmids #135012-135014). Guide RNA and ssODN sequences, as well as other primers used for PCR, are listed in Supplementary Table S1. Plasmid donor DNA constructs used pBlueScript KS- as a backbone. The homology arm DNA was amplified by PCR from the cell lines’ genomic DNA and cloned into the backbone using the restriction sites noted in Supplementary Table S1. The donor plasmids for PSMB6-YFP were described in [16]. The cassette containing Clover driven by a CMV promoter was amplified from pcDNA3-Clover (pcDNA3-Clover was a gift from Michael Lin (Addgene plasmid #40259; http://n2t.net/addgene:40259; RRID: Addgene_40259) [35]. Transfections were performed using JetPEI^®^ (Polyplus-transfection SA, Illkirch, France) or with polyethylenimine (PEI) 25 K (Polysciences) prepared at 1 mg/mL with a protocol like the commercial JetPEI reagent. Transfection conditions for co-editing were as follows: cells in 12 well dishes were transfected with 400 ng donor plasmid, 10 pmol ssODN for the rescue of TAFts, and 20 ng Flag-Cas9/sgRNA plasmids. These conditions were scaled as needed for other sized wells. For experiments comparing ts selection to unselected cells, two days post-transfection, cells were replated into two dishes, with one maintained at 37 °C and passaged to maintain a healthy culture over the course of the experiment (typically 3–4 weeks). The other plate was incubated at 39.5 °C and cells were not passaged, but the medium was changed.

### 4.4. SgRNA Design and Analysis of Editing

SgRNAs were designed, and off-target cutting assessed using the CRISPR design tool, Zhang lab, MIT [36], or the Desktop Genetics design tool (deskgen.com). Off-target cutting was also evaluated by CHOPCHOP v3 [37] and by the Synthego validation tool (https://design.synthego.com/#/validate; accessed 1 April 2021)(Supplementary Table S2). In the design of the guides used for this study, we looked for sgRNAs that produced a cut closest to the site of editing, as this has been shown to improve the efficiency of knock-in [38]. To prepare genomic DNA for PCR analysis, cell pellets from approximately 10^5^ cells were suspended in 18 μL 50 mM NaOH and heated at 95 °C for 10 min, followed by neutralization with 2 μL 1 M Tris-Cl pH 8. Primers listed in Supplementary Table S1 were used to amplify the respective fragments, and these were sequenced via Sanger sequencing (an automated dideoxy sequencing using a 3730 DNA Analyzer (ABI)) by the Weizmann Institute DNA Sequencing Unit. The Synthego ICE tool (inference of CRISPR edits) (https://ice.synthego.com/#/; accessed 1 April 2021) was used to deconvolute the Sanger sequencing results of edited clones. qPCR of genomic DNA was performed using the LightCycler480 (Roche), with PerfeCta^®^SYBR Green Fast Mix (QuantaBio, Beverly, MA, USA).

### 4.5. RNA Extraction and cDNA Preparation and Analysis

Total RNA was extracted using BIO TRI RNA reagent (Bio-Lab, Jerusalem, Israel). First-strand synthesis was performed using qScript Flex cDNA synthesis kit (QuantaBio) using a TAF1/TAF1L-specific 3′ primer. cDNA was used to amplify the TAF1/TAF1L fragment, and the PCR product was sequenced by Sanger sequencing.

### 4.6. Protein Gels and Immunoblotting

SDS-PAGE and immunoblotting were performed as previously described [39] using RIPA buffer (50 mM Tris-HCl pH 7.5, 150 mM NaCl, 1% Nonidet *P*-40 (*v/v*), 0.5% deoxycholate (*v/v*), 0.1% SDS (*w/v*)) supplemented with a cocktail of protease inhibitors (Apex Bio) for cell extract preparation. Antibodies used were monoclonal anti-β-tubulin, anti-β-actin, and anti-FLAG M2 (Sigma, St. Louis, MO, USA); anti-c-Abl K-12 (Santa Cruz Biotechnology, Santa Cruz, CA, USA). The polyclonal Living Colors antibody (Clontech) was used to detect SYFP and Clover. Horseradish peroxidase-conjugated secondary antibodies were from Jackson ImmunoResearch Laboratories, West Grove, PA. Enhanced chemiluminescence was performed with the EZ-ECL kit (Biological Industries, Kibbutz Beit Hemek, Israel), and signals were detected by the ImageQuant LAS 4000 (GE Healthcare, Piscataway, NJ, USA). Intensities of bands were quantified by the ImageQuant TL software. For comparison of multiple experiments, values within one experiment were normalized to a standard set at 1. “SEM” refers to the standard error of the mean.

### 4.7. Proteasome Activity Assay

The proteasomal activity was assessed in cytosolic cell extracts by measuring the hydrolysis of fluorogenic peptide substrates as described [40]. Z-LLE-AMC and Suc-LLVY-AMC (Biomol, Hamburg, Germany) were used for the measurement of caspase-like and chymotrypsin-like activities, respectively. Cellular extracts were incubated with the substrates in 96-well plates, and the release of the fluorescent free AMC group was measured by the infinite 200 multifunctional microplate reader (TECAN, Mannedorf, Switzerland).

### 4.8. Statistical Analysis

A Student’s *t*-test (two-sided, unequal variance), using GraphPad Prism, was performed to assess significance.

### 4.9. Flow Cytometry

Live cells were harvested and washed with PBS. For each measurement, 50–100,000 cells were collected by the BD LSRII flow cytometer (Becton Dickinson, Mountain View, CA, USA) and analyzed with the BD FACSDiva software (BD Biosciences, San Jose, CA, USA).

### 4.10. Figures

Figures were created with Biorender.com.

## 5. Patents

This manuscript describes inventions covered in the international patent application No. PCT/IL2020/051322 (YS and NR, inventors).

## Supplementary Materials

The following are available online at https://www.mdpi.com/article/10.3390/ijms22073741/s1.

## References

[1] Cong L., Ran F.A., Cox D., Lin S., Barretto R., Habib N., Hsu P.D., Wu X., Jiang W., Marraffini L.A. (2013). Multiplex genome engineering using CRISPR/Cas systems. Science.

[2] Barrangou R., Doudna J.A. (2016). Applications of CRISPR technologies in research and beyond. Nat. Biotechnol..

[3] Komor A.C., Badran A.H., Liu D.R. (2017). CRISPR-Based Technologies for the Manipulation of Eukaryotic Genomes. Cell.

[4] Mali P., Yang L., Esvelt K.M., Aach J., Guell M., DiCarlo J.E., Norville J.E., Church G.M. (2013). RNA-guided human genome engineering via Cas9. Science.

[5] Yang H., Wang H., Shivalila C.S., Cheng A.W., Shi L., Jaenisch R. (2013). One-step generation of mice carrying reporter and conditional alleles by CRISPR/Cas-mediated genome engineering. Cell.

[6] Yeh C.D., Richardson C.D., Corn J.E. (2019). Advances in genome editing through control of DNA repair pathways. Nat. Cell Biol..

[7] Yang H., Ren S., Yu S., Pan H., Li T., Ge S., Zhang J., Xia N. (2020). Methods Favoring Homology-Directed Repair Choice in Response to CRISPR/Cas9 Induced-Double Strand Breaks. Int. J. Mol. Sci..

[8] Chu V.T., Weber T., Wefers B., Wurst W., Sander S., Rajewsky K., Kuhn R. (2015). Increasing the efficiency of homology-directed repair for CRISPR-Cas9-induced precise gene editing in mammalian cells. Nat. Biotechnol..

[9] Maruyama T., Dougan S.K., Truttmann M.C., Bilate A.M., Ingram J.R., Ploegh H.L. (2015). Increasing the efficiency of precise genome editing with CRISPR-Cas9 by inhibition of nonhomologous end joining. Nat. Biotechnol..

[10] Song J., Yang D., Xu J., Zhu T., Chen Y.E., Zhang J. (2016). RS-1 enhances CRISPR/Cas9- and TALEN-mediated knock-in efficiency. Nat. Commun..

[11] Gutschner T., Haemmerle M., Genovese G., Draetta G.F., Chin L. (2016). Post-translational Regulation of Cas9 during G1 Enhances Homology-Directed Repair. Cell Rep..

[12] Nihongaki Y., Kawano F., Nakajima T., Sato M. (2015). Photoactivatable CRISPR-Cas9 for optogenetic genome editing. Nat. Biotechnol..

[13] Tran N.T., Bashir S., Li X., Rossius J., Chu V.T., Rajewsky K., Kuhn R. (2019). Enhancement of Precise Gene Editing by the Association of Cas9 With Homologous Recombination Factors. Front. Genet..

[14] Jayavaradhan R., Pillis D.M., Goodman M., Zhang F., Zhang Y., Andreassen P.R., Malik P. (2019). CRISPR-Cas9 fusion to dominant-negative 53BP1 enhances HDR and inhibits NHEJ specifically at Cas9 target sites. Nat. Commun..

[15] Charpentier M., Khedher A.H.Y., Menoret S., Brion A., Lamribet K., Dardillac E., Boix C., Perrouault L., Tesson L., Geny S. (2018). CtIP fusion to Cas9 enhances transgene integration by homology-dependent repair. Nat. Commun..

[16] Reuven N., Adler J., Broennimann K., Myers N., Shaul Y. (2019). Recruitment of DNA Repair MRN Complex by Intrinsically Disordered Protein Domain Fused to Cas9 Improves Efficiency of CRISPR-Mediated Genome Editing. Biomolecules.

[17] Li X.L., Li G.H., Fu J., Fu Y.W., Zhang L., Chen W., Arakaki C., Zhang J.P., Wen W., Zhao M. (2018). Highly efficient genome editing via CRISPR-Cas9 in human pluripotent stem cells is achieved by transient BCL-XL overexpression. Nucleic Acids Res..

[18] Thomas M.C., Chiang C.M. (2006). The general transcription machinery and general cofactors. Crit. Rev. Biochem. Mol. Biol..

[19] Antonova S.V., Boeren J., Timmers H.T.M., Snel B. (2019). Epigenetics and transcription regulation during eukaryotic diversification: The saga of TFIID. Genes Dev..

[20] Hayashida T., Sekiguchi T., Noguchi E., Sunamoto H., Ohba T., Nishimoto T. (1994). The CCG1/TAFII250 gene is mutated in thermosensitive G1 mutants of the BHK21 cell line derived from golden hamster. Gene.

[21] Wang P.J., Page D.C. (2002). Functional substitution for TAF(II)250 by a retroposed homolog that is expressed in human spermatogenesis. Hum. Mol. Genet..

[22] Thul P.J., Akesson L., Wiking M., Mahdessian D., Geladaki A., Ait Blal H., Alm T., Asplund A., Bjork L., Breckels L.M. (2017). A subcellular map of the human proteome. Science.

[23] Landry J.J., Pyl P.T., Rausch T., Zichner T., Tekkedil M.M., Stutz A.M., Jauch A., Aiyar R.S., Pau G., Delhomme N. (2013). The genomic and transcriptomic landscape of a HeLa cell line. G3 Genes Genomes Genet..

[24] Arendt C.S., Hochstrasser M. (1997). Identification of the yeast 20S proteasome catalytic centers and subunit interactions required for active-site formation. Proc. Natl. Acad. Sci. USA.

[25] Heinemeyer W., Fischer M., Krimmer T., Stachon U., Wolf D.H. (1997). The active sites of the eukaryotic 20 S proteasome and their involvement in subunit precursor processing. J. Biol. Chem..

[26] Sehnal D., Rose A.S., Koča J., Burley S.K., Velankar S. (2018). Mol*: Towards a common library and tools for web molecular graphics. Workshop on Molecular Graphics and Visual Analysis of Molecular Data.

[27] Schrader J., Henneberg F., Mata R.A., Tittmann K., Schneider T.R., Stark H., Bourenkov G., Chari A. (2016). The inhibition mechanism of human 20S proteasomes enables next-generation inhibitor design. Science.

[28] Komor A.C., Kim Y.B., Packer M.S., Zuris J.A., Liu D.R. (2016). Programmable editing of a target base in genomic DNA without double-stranded DNA cleavage. Nature.

[29] Anzalone A.V., Randolph P.B., Davis J.R., Sousa A.A., Koblan L.W., Levy J.M., Chen P.J., Wilson C., Newby G.A., Raguram A. (2019). Search-and-replace genome editing without double-strand breaks or donor DNA. Nature.

[30] Benitez E.K., Lomova Kaufman A., Cervantes L., Clark D.N., Ayoub P.G., Senadheera S., Osborne K., Sanchez J.M., Crisostomo R.V., Wang X. (2020). Global and Local Manipulation of DNA Repair Mechanisms to Alter Site-Specific Gene Editing Outcomes in Hematopoietic Stem Cells. Front. Genome Ed..

[31] Agudelo D., Duringer A., Bozoyan L., Huard C.C., Carter S., Loehr J., Synodinou D., Drouin M., Salsman J., Dellaire G. (2017). Marker-free coselection for CRISPR-driven genome editing in human cells. Nat. Methods.

[32] Li S., Akrap N., Cerboni S., Porritt M.J., Wimberger S., Lundin A., Moller C., Firth M., Gordon E., Lazovic B. (2021). Universal toxin-based selection for precise genome engineering in human cells. Nat. Commun..

[33] Yan N., Sun Y., Fang Y., Deng J., Mu L., Xu K., Mymryk J.S., Zhang Z. (2019). A Universal Surrogate Reporter for Efficient Enrichment of CRISPR/Cas9-Mediated Homology-Directed Repair in Mammalian Cells. Mol. Ther. Nucleic Acids.

[34] Liao S., Tammaro M., Yan H. (2015). Enriching CRISPR-Cas9 targeted cells by co-targeting the HPRT gene. Nucleic Acids Res..

[35] Lam A.J., St-Pierre F., Gong Y., Marshall J.D., Cranfill P.J., Baird M.A., McKeown M.R., Wiedenmann J., Davidson M.W., Schnitzer M.J. (2012). Improving FRET dynamic range with bright green and red fluorescent proteins. Nat. Methods.

[36] Hsu P.D., Scott D.A., Weinstein J.A., Ran F.A., Konermann S., Agarwala V., Li Y., Fine E.J., Wu X., Shalem O. (2013). DNA targeting specificity of RNA-guided Cas9 nucleases. Nat. Biotechnol..

[37] Labun K., Montague T.G., Krause M., Torres Cleuren Y.N., Tjeldnes H., Valen E. (2019). CHOPCHOP v3: Expanding the CRISPR web toolbox beyond genome editing. Nucleic Acids Res..

[38] Yang L., Guell M., Byrne S., Yang J.L., De Los Angeles A., Mali P., Aach J., Kim-Kiselak C., Briggs A.W., Rios X. (2013). Optimization of scarless human stem cell genome editing. Nucleic Acids Res..

[39] Levy D., Adamovich Y., Reuven N., Shaul Y. (2007). The Yes-associated protein 1 stabilizes p73 by preventing Itch-mediated ubiquitination of p73. Cell. Death Differ..

[40] Kisselev A.F., Goldberg A.L. (2005). Monitoring activity and inhibition of 26S proteasomes with fluorogenic peptide substrates. Methods Enzym..

