# Supplementary material for: CRISPR Co-Editing Strategy for Scarless Homology-Directed Genome Editing
Source: Int J Mol Sci. 2021 Apr 3;22(7):3741. doi: 10.3390/ijms22073741 (PMC8038335; doi:10.3390/ijms22073741)

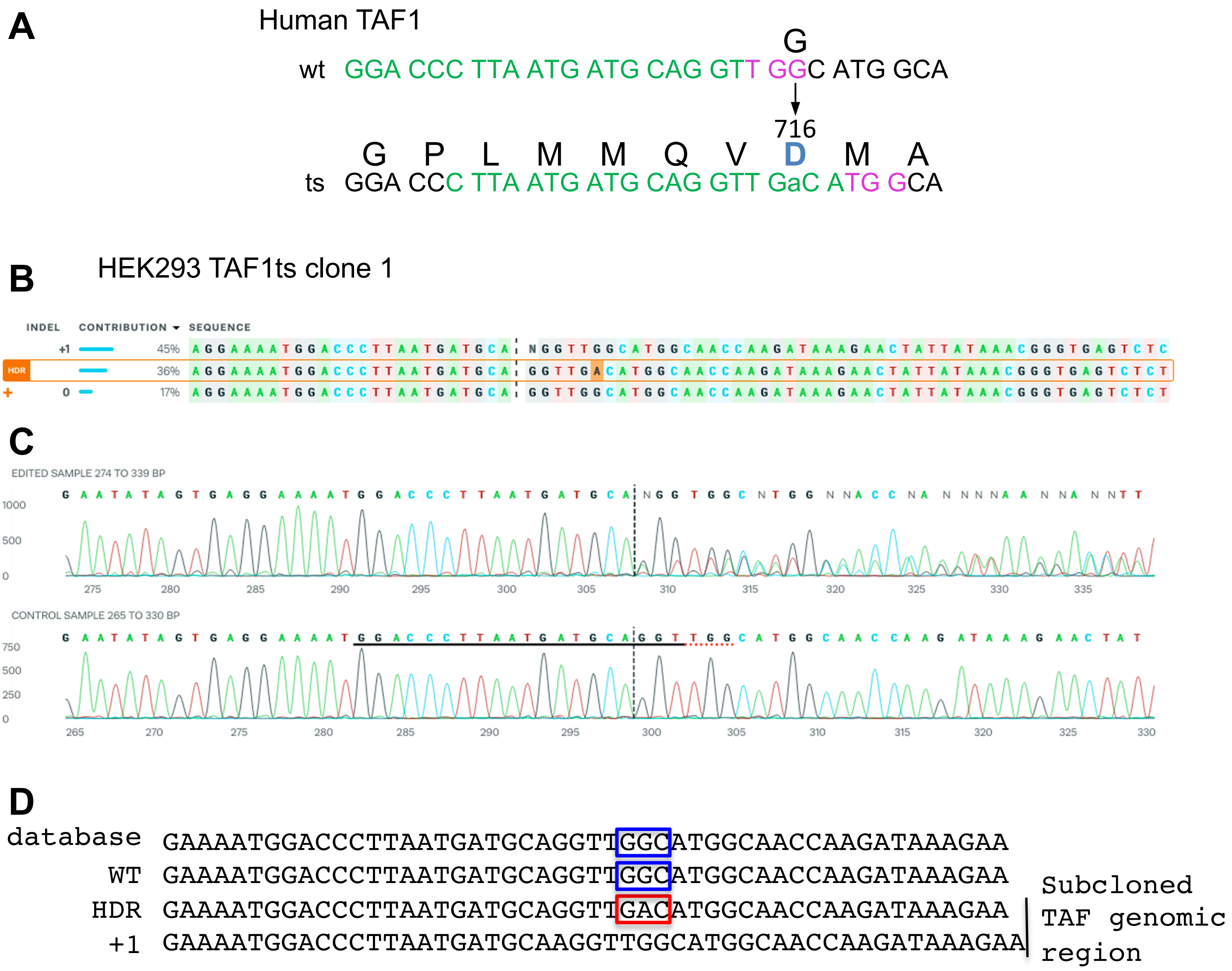

Supplementary Figure S1. Genotypic and phenotypic characterization of HEK293 TAF1 ts cells. (A) sgRNAs used to target human TAF1. The guide RNAs are shown in green, with the PAM sequences in pink. The guide used to create the TAF1 ts cells (G716D) is indicated on the upper wt sequence. The guide used to target the TAF1ts cells is indicated on the lower, ts sequence. **(B, C, D) Sequences of the TAF1 alleles in HEK293 TAF1ts clones 1.** The 554 bp fragment from the ts clone was amplified, sequenced, and the results were analyzed by using the Synthego ICE tool (Synthego Performance Analysis, ICE Analysis. 2019. v2. Synthego) **(B)**. The results show the planned G716D mutation (marked HDR), as well as +1 insertion. The insertion causes frame-shifting and early termination (10 amino acid addition, then stop codon). **(C)** Sanger sequencing results of the edited and control samples. The guide sequence is underlined, the PAM underlined with a dotted red line, and the location of the Cas9 cleavage indicated by the vertical dashed line. **(D)** In order to experimentally separate sequences of CRISPR TAFts clones, PCR on the respective genomic regions was done with the addition of BamHI and XhoI sites and cloned into pCDNA. Sequencing of 6 clones demonstrated HDR in 4 clones and +1 insertion in 2 clones, thus confirming the results of ICE analysis. The third allele of TAF1 in this clone had a large out-of-frame insertion of 174 bp downstream from the cut site, which was not well analyzed by ICE analysis of the 554bp fragment. Here (B), it was detected as a small contribution of wt sequence. Subcloning and sequencing of the 554bp and 728bp PCR products revealed the sequence of this allele, confirming that it will lead to a knocked-out gene product.

A HEK293 TAF1ts clone 11

B

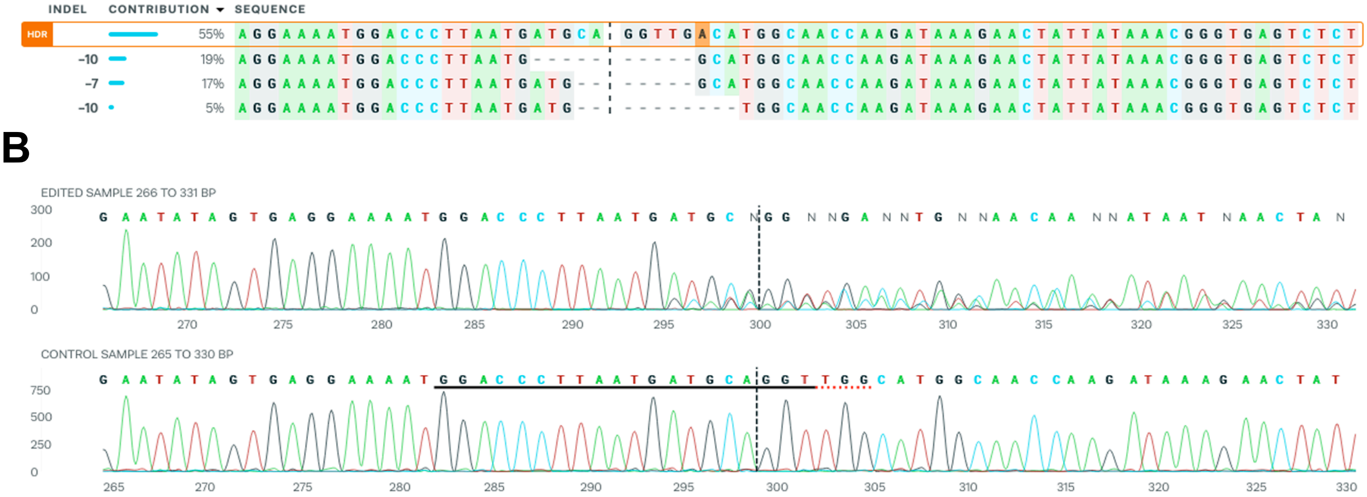

C

```
database GAAAATGGACCCTTAATGATGCAGGTTGGCATGGCAACCAAGATAAAGAA
      WT GAAAATGGACCCTTAATGATGCAGGTTGGCATGGCAACCAAGATAAAGAA
     HDR GAAAATGGACCCTTAATGATGCAGGTTGACATGGCAACCAAGATAAAGAA| subcloned
     -10 GAAAATGGACCCTTAATG::::::::::GCATGGCAACCAAGATAAAGAA| TAF genomic
      -7 GAAAATGGACCCTTAATGATG:::::::GCATGGCAACCAAGATAAAGAA| region
```

Supplementary Figure S2. TAF1 sequencing analysis of HEK293 TAF1 ts cells (clone11). The 554 bp fragment from the ts clone was amplified, sequenced, and the results were analyzed by using the Synthego ICE tool (Synthego Performance Analysis, ICE Analysis. 2019. v2. Synthego) **(A)**. The results show the planned G716D mutation (marked HDR), as well as -7 and -10 deletions. Another sequence suggestion is likely due to noisiness of the sequencing results. Both deletions cause frame-shifting and early termination (5-6 amino acid addition, then stop codon). **(B)** Sanger sequencing results of the edited and control samples. The guide sequence is underlined, the PAM underlined with a dotted red line, and the location of the Cas9 cleavage indicated by the vertical dashed line. **(C)** In order to experimentally separate sequences of CRISPR TAFts clones, PCR on the respective genomic regions was done with the addition of BamHI and XhoI sites and cloned into pCDNA. Sequencing of 11 clones demonstrated HDR in 6 clones, -10 deletion in 4 clones, and -7 deletion in 1 clone, thus confirming the results of ICE analysis

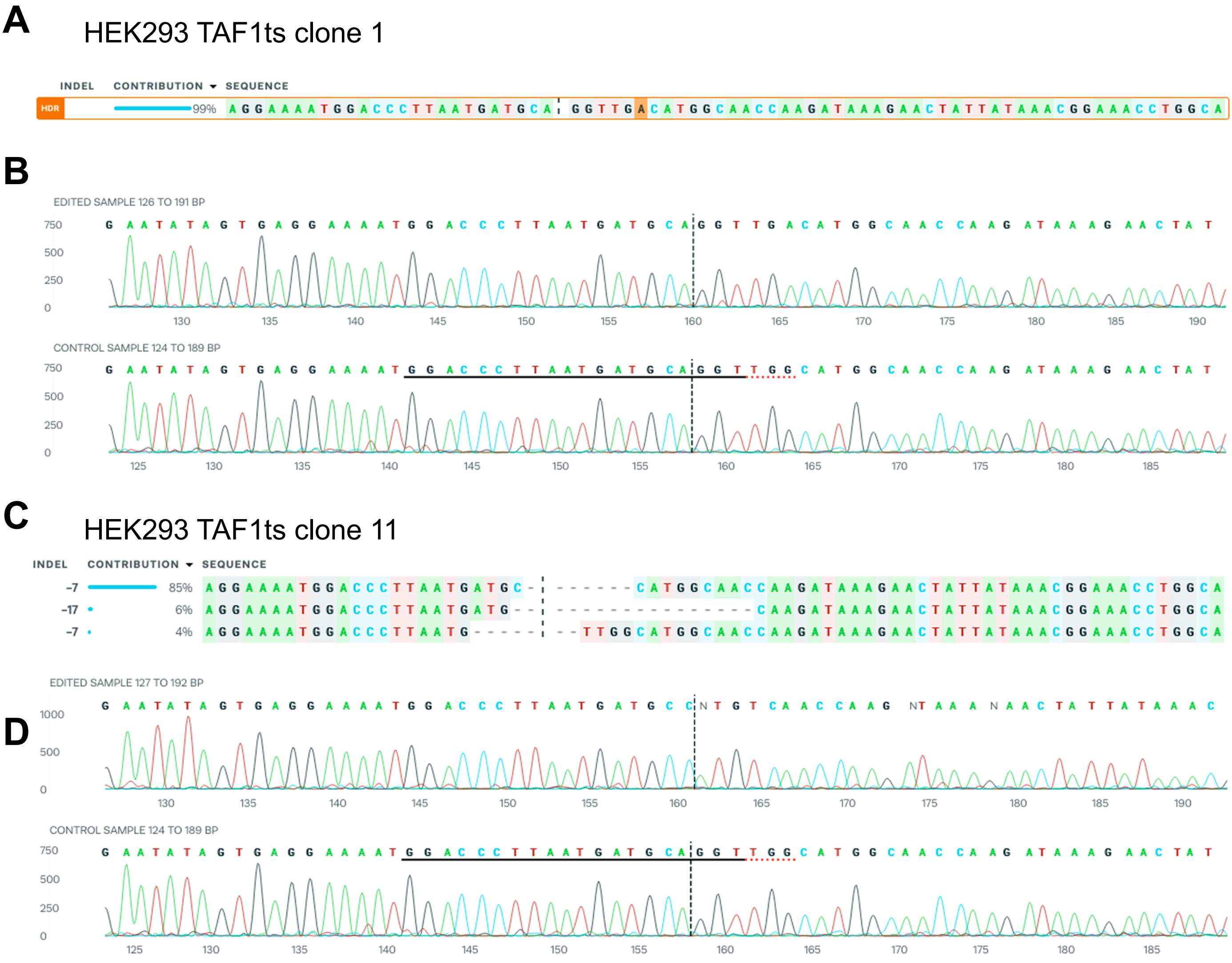

Supplementary Figure S3. TAF1L sequencing analysis of HEK293 TAF1 ts cells (clone1 and clone 11). An 879bp fragment from the ts clone was amplified, sequenced, and the results were analyzed by using the Synthego ICE tool (Synthego Performance Analysis, ICE Analysis. 2019. v2. Synthego) **(A)**. The results for HEK293 TAFts clone 1 show the TAF ts mutation. **(B)** Sanger sequencing results of the edited and control samples. The guide sequence is underlined, the PAM underlined with a dotted red line, and the location of the Cas9 cleavage indicated by the vertical dashed line. **(C)** The results for HEK293 TAFts clone 11 show -7 and -17 deletions. **(D)** Sanger sequencing results are presented, as in (B).

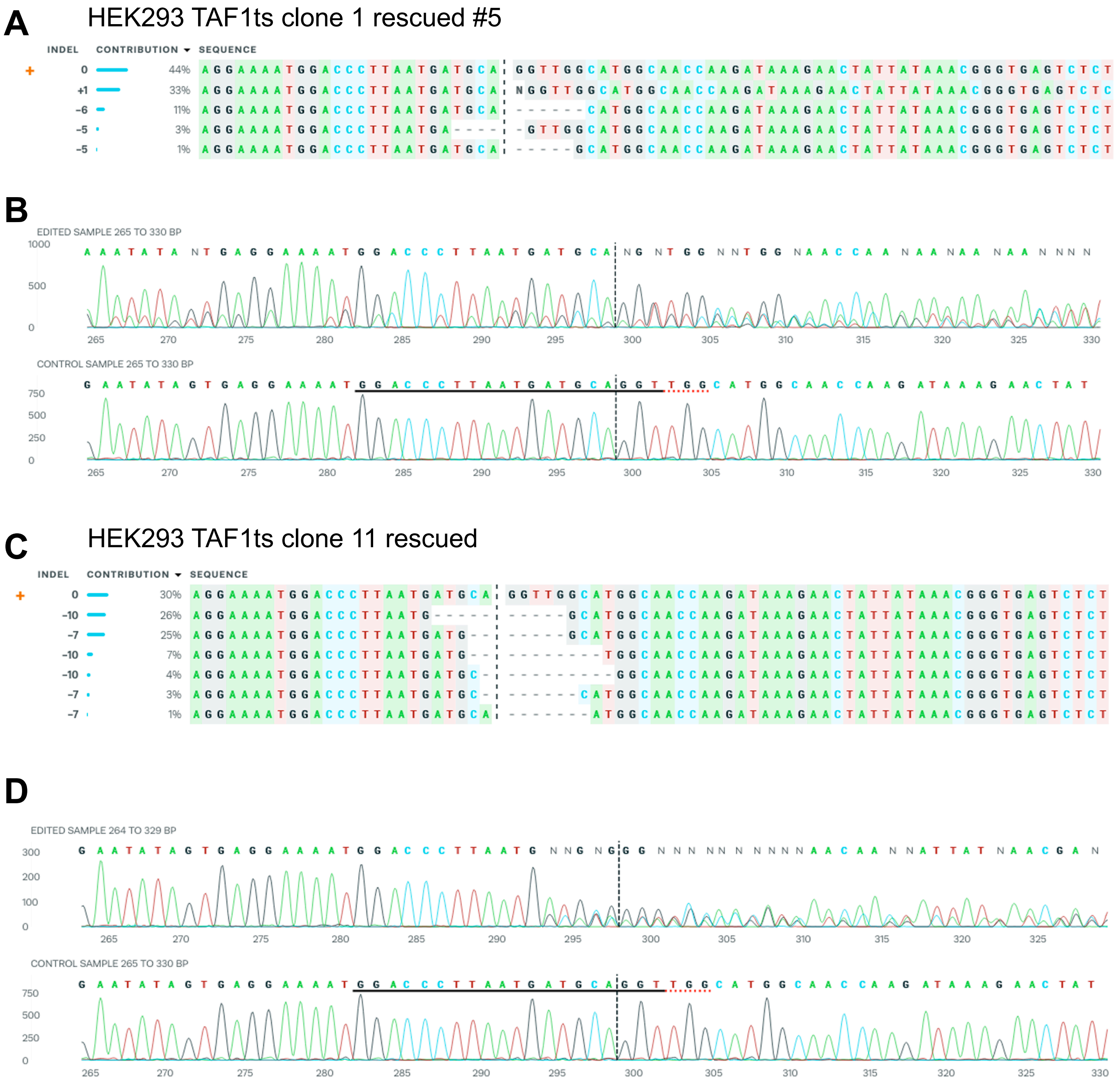

Supplementary Figure S4. TAF1 sequencing analysis of rescued clones of 293 TAF1 ts cells (clones 1 and 11). (A,B) HEK293 TAF1 ts cells (clone1 rescued #5). The 554 bp fragment from the corrected clone was amplified, sequenced, and the results were analyzed by using the Synthego ICE tool (Synthego Performance Analysis, ICE Analysis. 2019. v2. Synthego) **(A)**. The results show the complete correction of G716D mutation to the WT (marked 0), and a previously detected +1 insertion. The other sequence suggestions are likely due to noisiness of the sequencing results. **(B)** Sanger sequencing results of the edited and control samples. The guide sequence is underlined, the PAM underlined with a dotted red line, and the location of the Cas9 cleavage indicated by the vertical dashed line. **(C,D) HEK293 TAF1 ts cells (clone 11 rescued) (C)** The results from the ICE analysis show complete correction of G716D mutation back to the WT (marked 0), and the previously detected -7 and -10 deletions. The other sequence suggestions are likely due to noisiness of the sequencing results. **(D)** Sanger sequencing results of the edited and control samples.

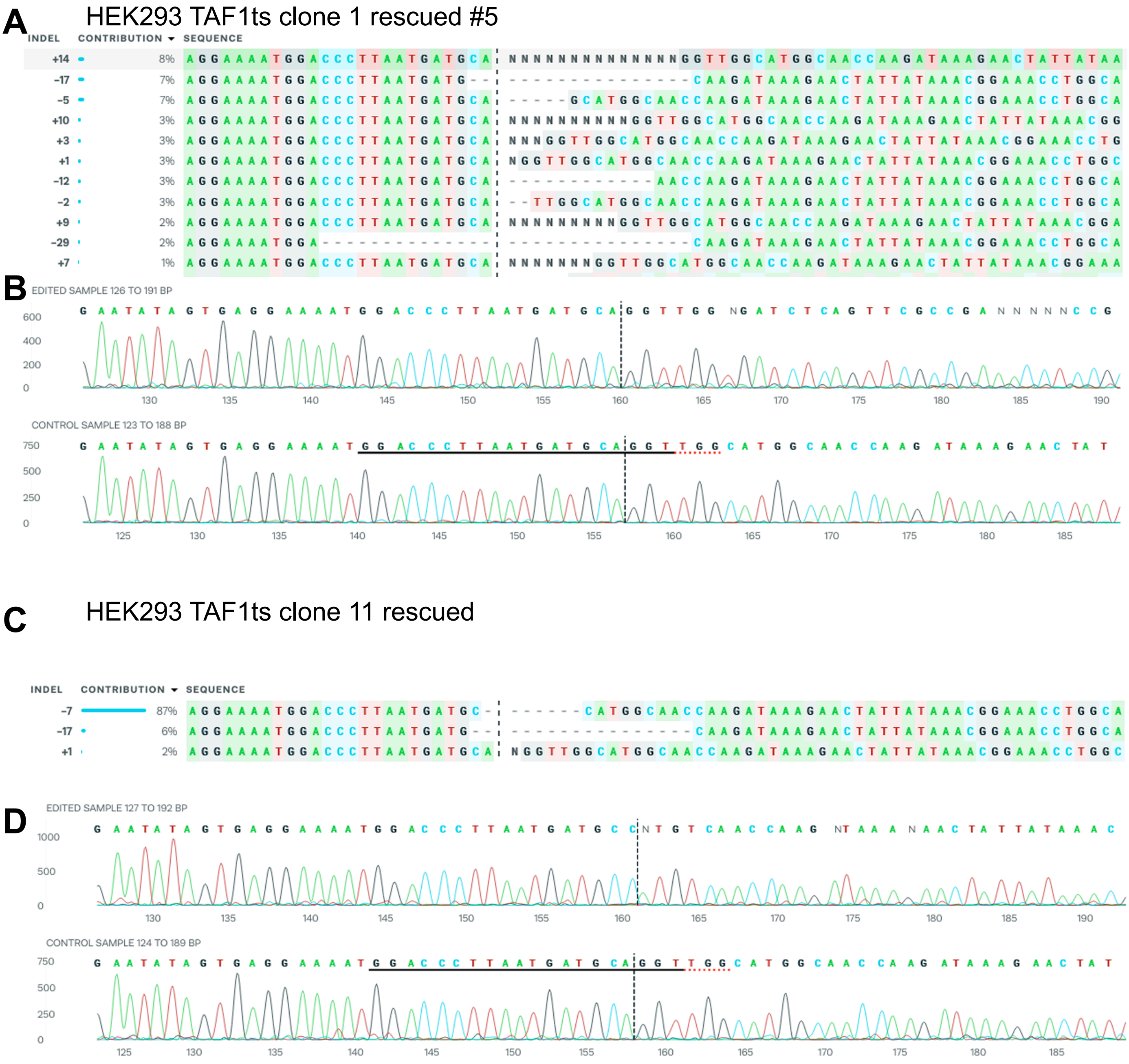

Supplementary Figure S5. TAF1L sequencing analysis of rescued clones of 293 TAF1 ts cells (clones 1 and 11). (A,B) HEK293 TAF1 ts cells (clone1 rescued #5). The 879 bp TAF1L fragment from the rescued clone was amplified, sequenced, and the results were analyzed by using the Synthego ICE tool (Synthego Performance Analysis, ICE Analysis. 2019. v2. Synthego) **(A)**. The results show a number of suggested sequences, likely due to the difficulty for the software to identify the large indel. **(B)** Sanger sequencing results of the edited and control samples. The guide sequence is underlined, the PAM underlined with a dotted red line, and the location of the Cas9 cleavage indicated by the vertical dashed line. **(C,D) HEK293 TAF1 ts cells (clone 11 rescued) (C)** The results from the ICE analysis show the previously detected -7 and -17 deletions. **(D)** Sanger sequencing results of the edited and control samples.

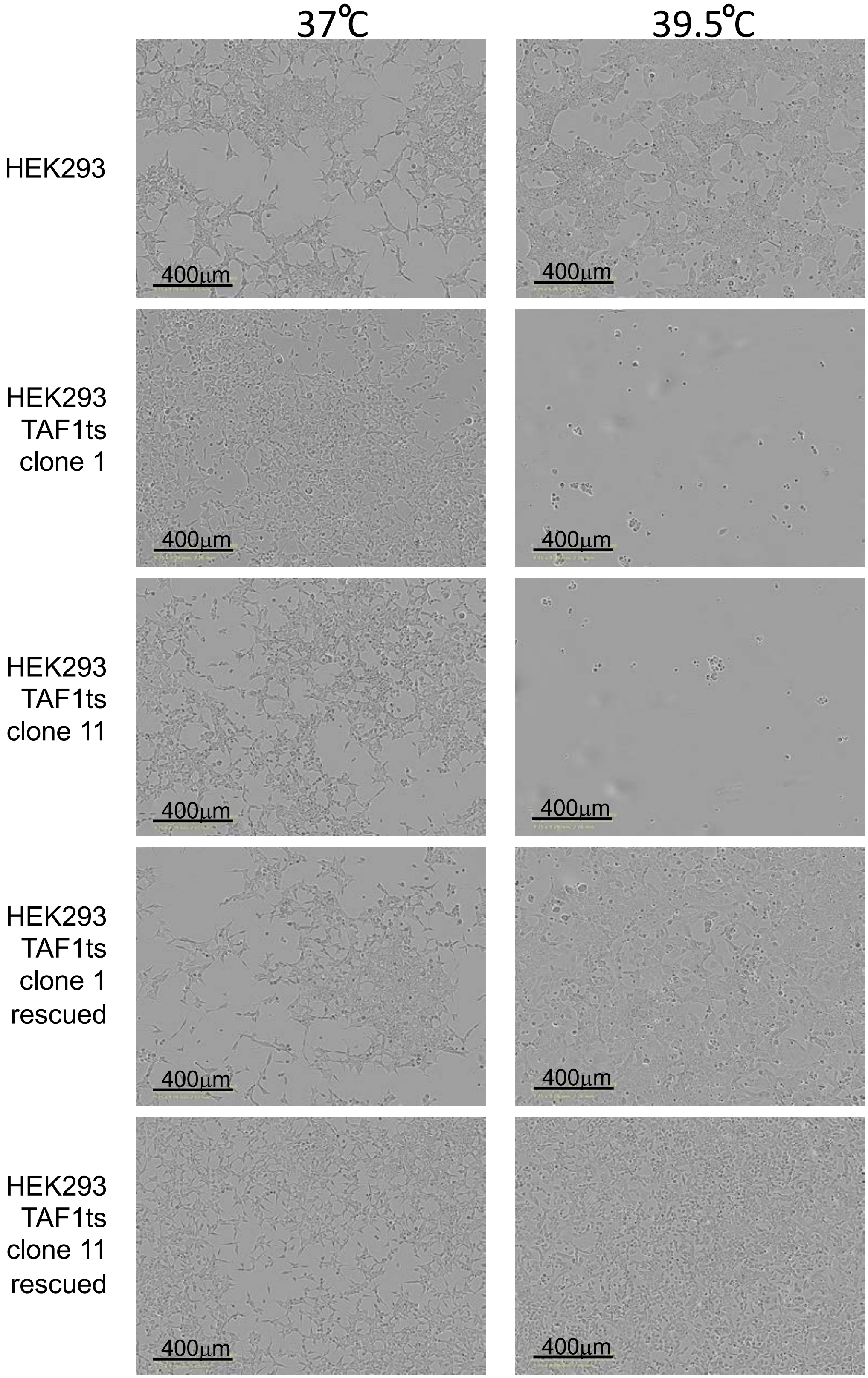

Supplementary Figure S6. Morphology of HEK293 naïve, TAF1ts, and TAF1ts rescued. HEK293 naïve, TAF1ts clones 1 and 11, and CRISPR-corrected clones of the ts cells were plated in 24 well plates at 14,000 cells/well. Cells were photographed by the Incucyte®SX1 Live-Cell Analysis System at 10x magnification. Pictures of cells growing exponentially at 37℃ 2-3 days post-plating are shown. Cells growing at 39.5 ℃ were photographed 5 days post-plating.

A

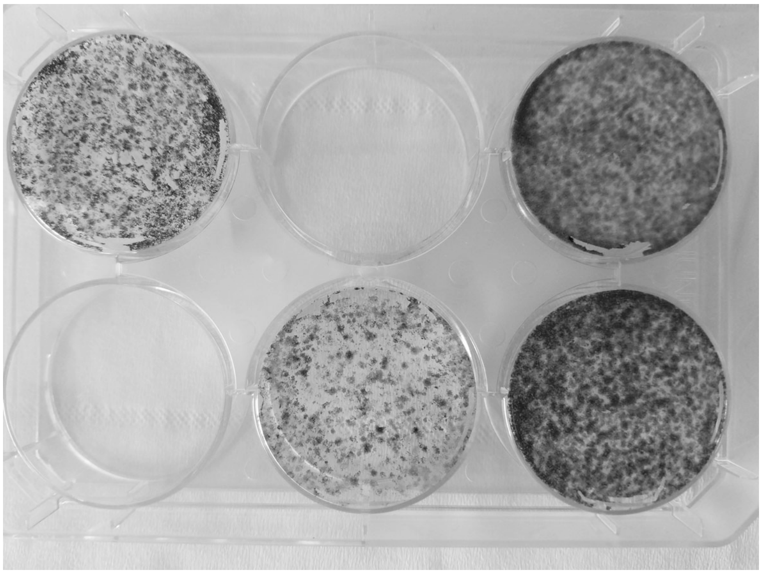

B

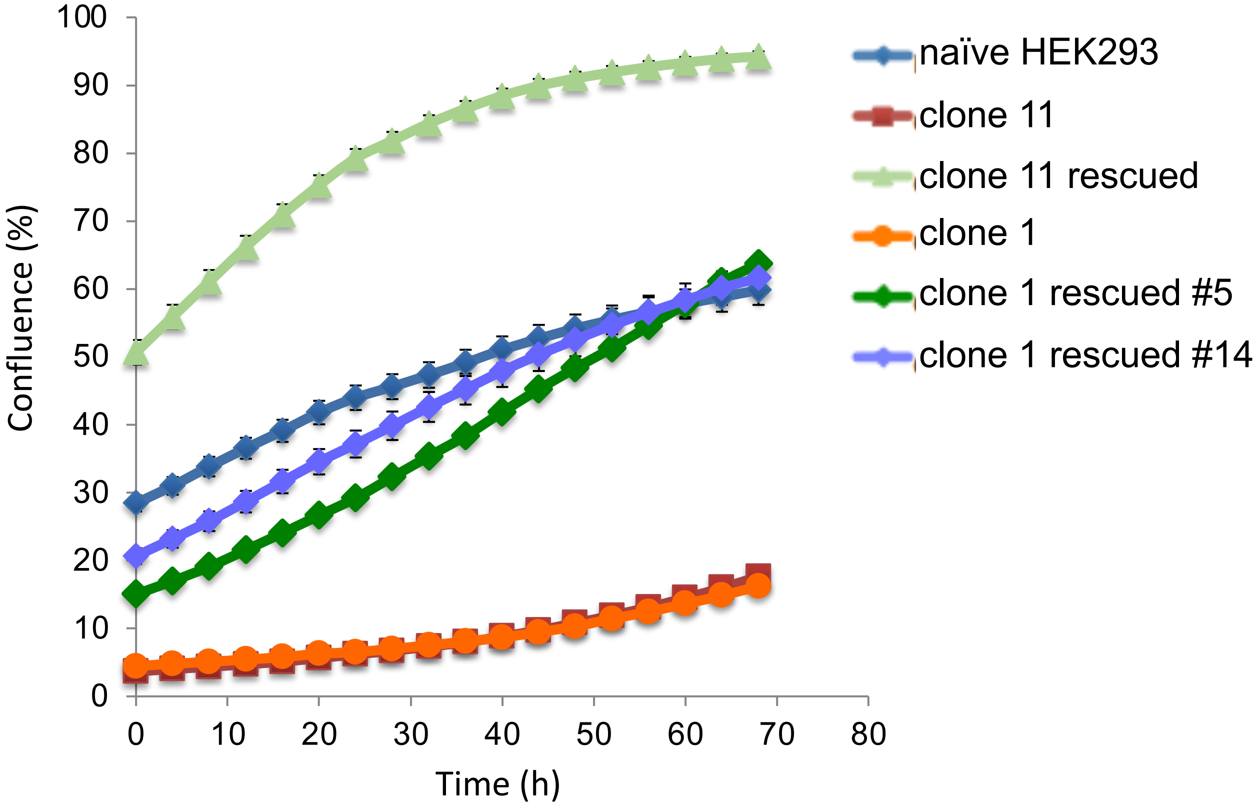

Supplementary Figure S7. Growth of HEK293 TAF1 ts and rescued clones at 39.5ºC and 37ºC. (A) Growth at 39.5ºC. HEK293 naïve, TAF1ts clones 1 and 11, and ts rescued clones were plated at 14,000 cells/well and incubated 7 days at 39.5ºC. Cells were stained with crystal violet. Two isolates of rescued clone 1 are shown (#5 and #14).**(B) Growth at 37ºC.** Cells were plated in quadruplicate in 24 well dishes at 14,000 cells/well and grown at 37ºC. Two days after plating, cells were photographed by the Incucyte®SX1 Live-Cell Analysis System at 10x magnification, 25 images per well, every 2h. Percent confluence was calculated by the Incucyte software, and was plotted vs. time.

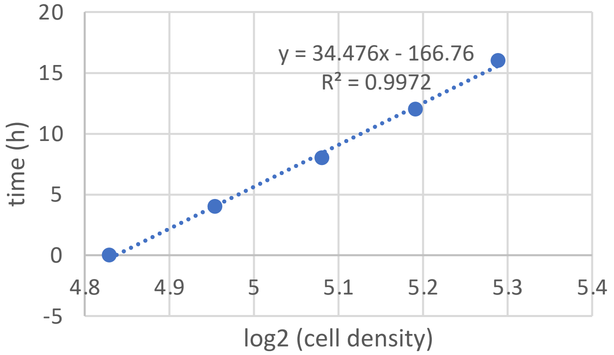

HEK293: 34.5 h

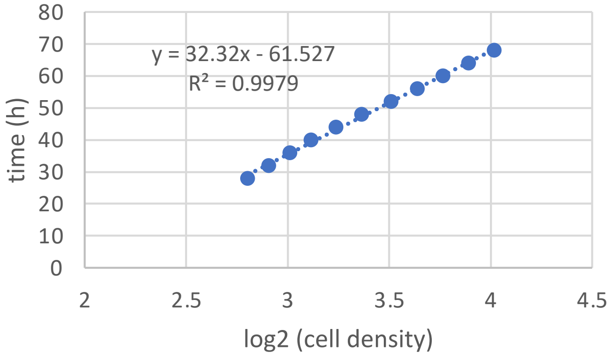

TAFts clone1: 32.3 h

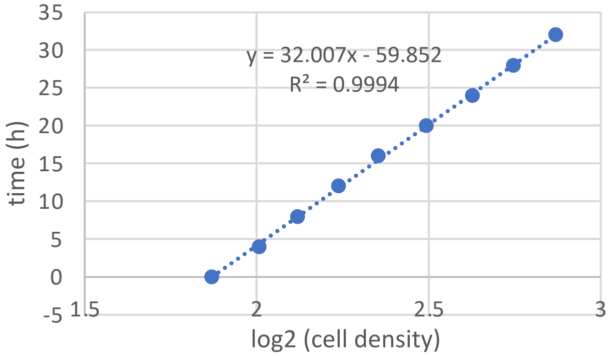

TAFts clone11: 32.0 h

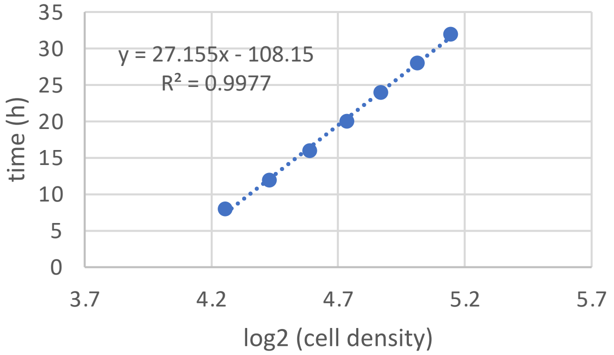

TAFts clone1 rescued: 27.2 h

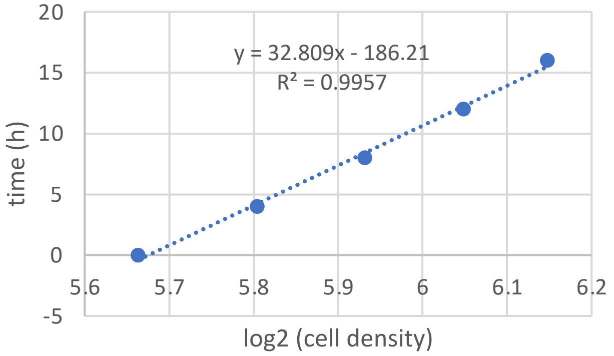

TAFts clone11 rescued: 32.8 h

Supplementary Figure S8. Doubling time calculation of naïve, ts, and ts rescued cells. HEK293 naïve, TAF1ts clones 1 and 11, and CRISPR-rescued clones of the ts cells were plated in quadruplicate in 24 well plates at 14,000 cells/well. Cells were photographed by the Incucyte®SX1 Live-Cell Analysis System at 10x magnification, 25 images per well, every 2h. Percent confluence was calculated by the Incucyte analysis software. Log2 of the confluence was plotted vs time for cells in exponential growth, with the slope of the line being the doubling time of the cells. The calculated doubling time is indicated below each graph.

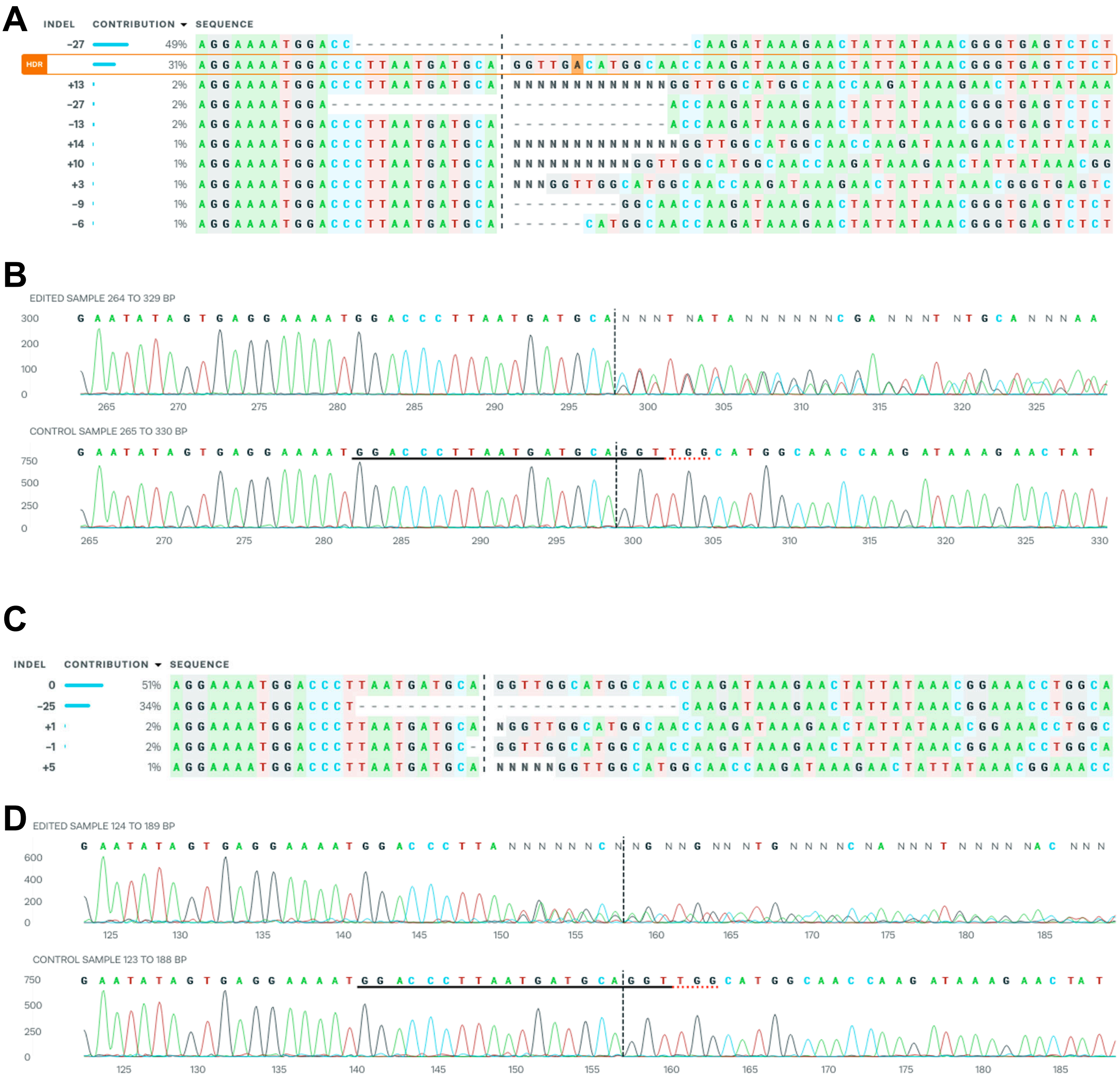

Supplementary Figure S9. TAF1 and TAF1L sequencing analysis of HeLa TAF1 G716D ts cells. (A) The 554 bp TAF1 fragment from the ts clone was amplified, sequenced, and the results were analyzed by using the Synthego ICE tool (Synthego Performance Analysis, ICE Analysis. 2019. v2. Synthego). The results show the planned G716D mutation (marked HDR) as well as large -27 deletion including the G716 position. The other sequence suggestions are likely due to noisiness of the sequencing results. **(B)** Sanger sequencing results of the edited and control samples. The guide sequence is underlined, the PAM underlined with a dotted red line, and the location of the Cas9 cleavage indicated by the vertical dashed line. **(C)** The 879 bp TAF1L fragment was analyzed as in (A). **(D)** Sanger sequencing results of the edited and control TAF1L samples, as in (B).

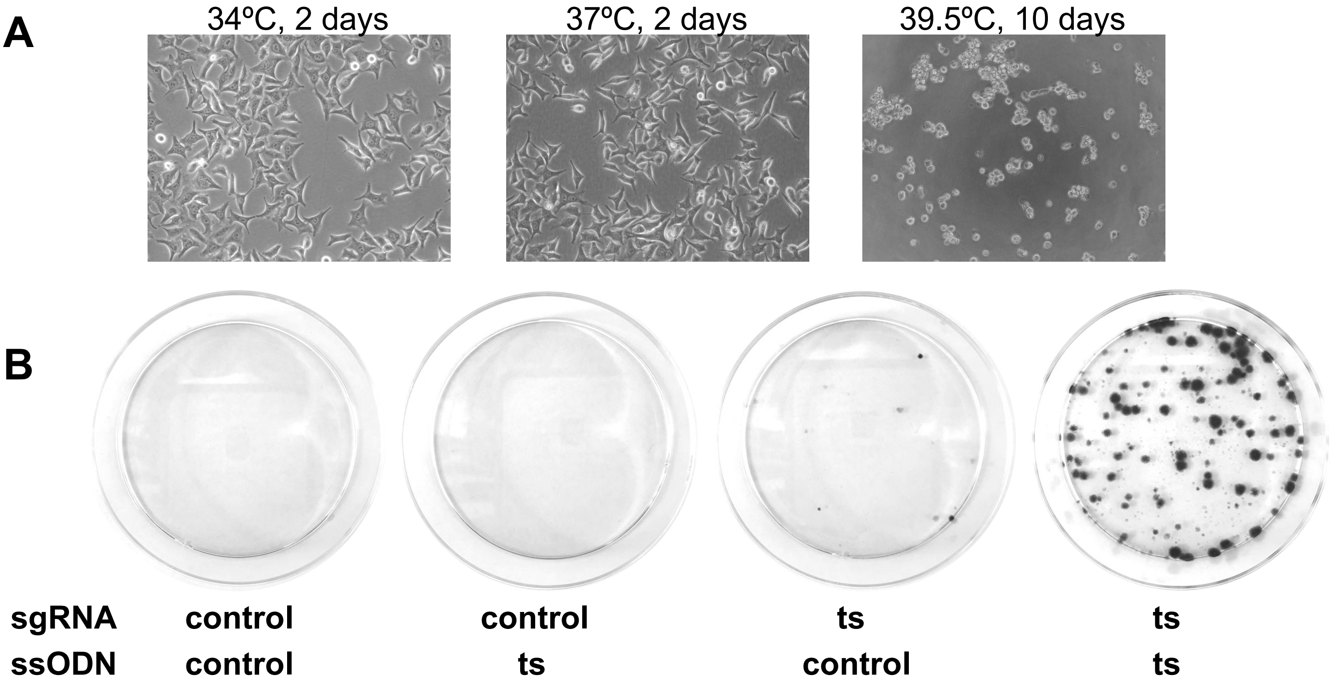

Supplementary Figure S10. Growth of HeLa TAF1 ts cells and rescue of the ts mutation. (A) HeLa TAF1 ts were grown at 34ºC, 37ºC and 39.5ºC, and photgraphed two or ten days post-plating, as indicated. **(B)** HeLa TAF1 ts cells were transfected with Cas9/sgRNA-encoding plasmids and ssODN. Two days post-transfection, cells were replated, and transferred the next day to 39.5 °C. 18 days later, colonies were stained with crystal violet.

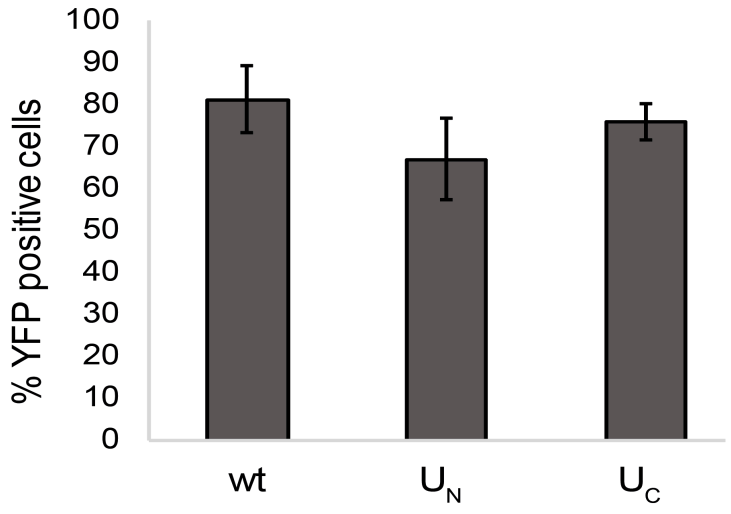

Supplementary Figure S11. Co-editing of PSMB6-YFP in HEK293 TAF1 ts cells with MRN-recruiting Cas9 constructs. HEK293 TAF1 ts cells were transfected as described in Figure 4B, in quadruplicates, using either wt Cas9, or the MRN-recruiting Cas9 constructs U_N and U_C. Heat-selected colonies were analyzed for YFP expression, and the percent of YFP positive colonies out of total heat-resistant colonies is summarized. N=4, error bars represent SEM.

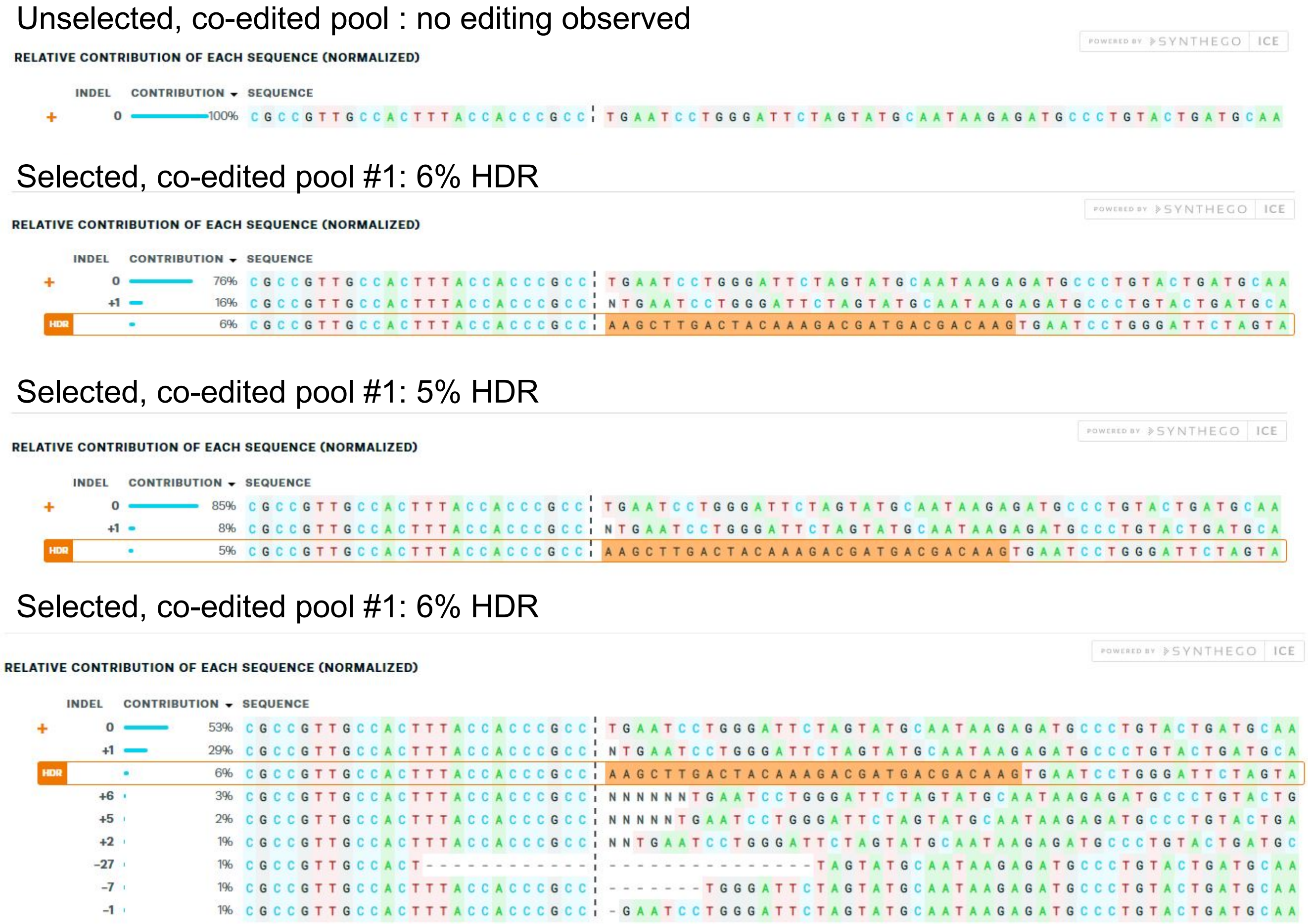

Supplementary Figure S12. Synthego ICE analysis of PSMB6-Flag edited cells. HEK293 TAF1 ts cells were transfected as described in Figure 4D. The 554 bp fragment from pools of unselected selected was amplified, sequenced, and the results were analyzed by using the Synthego ICE tool. Results from three independent pools of selected cells and one unselected are shown. The orange + indicates wt sequence, +1 indicates a 1nt insertion, HDR shows the inserted Flag tag.

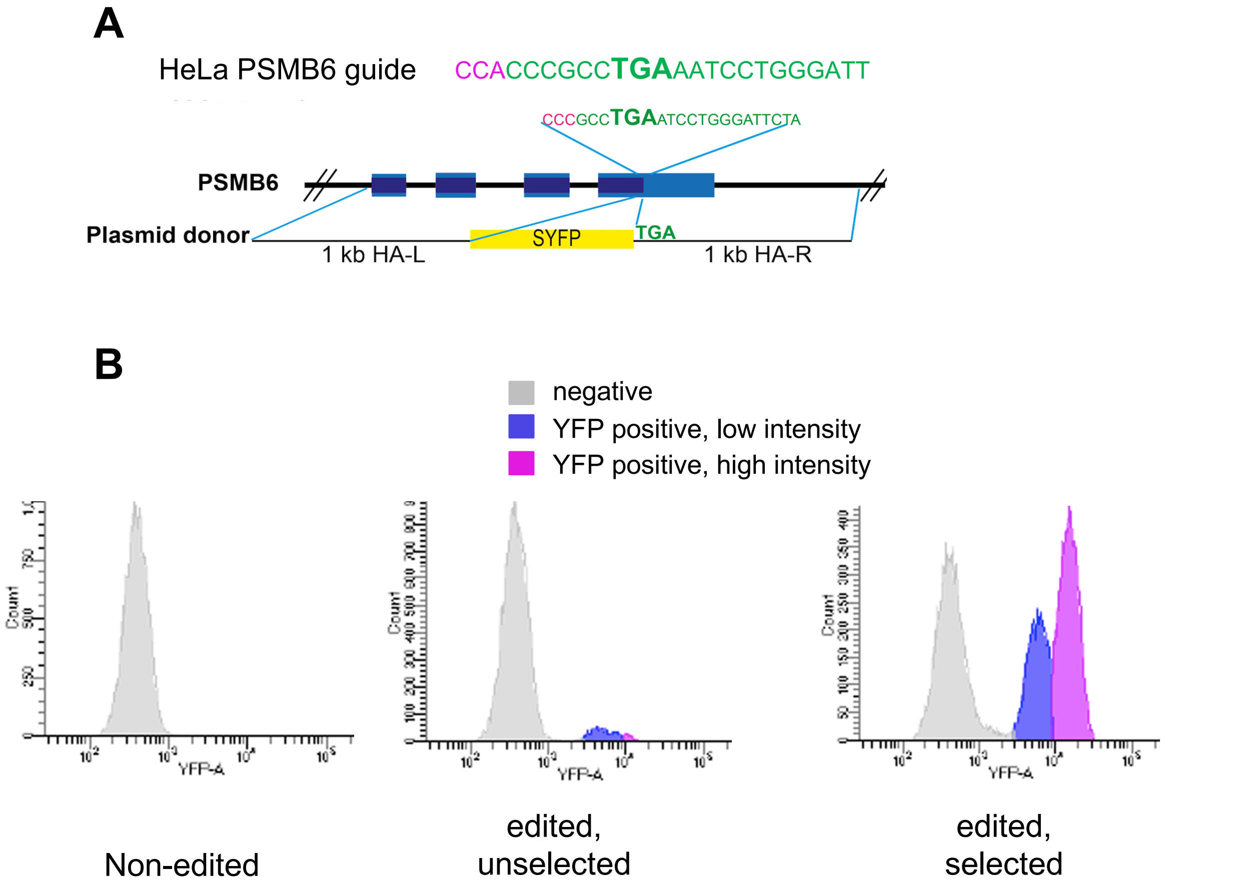

Supplementary Figure S13.PSMB6-YFP editing of HeLa cells. (A) Guide RNA used for editing HeLa TAF1ts. The original guide and the schematic for the PSMB6-YFP donor plasmid are shown below. (**B) FACS of HeLa ts co-edited for PSMB6-YFP.** HeLa ts cells co-edited for PSMB6-YFP as described in Figure 5 were analyzed by FACS with 30,000 cells per point in triplicates. FACS histogram of YFP positive cells showing two separate populations of low and high intensity.

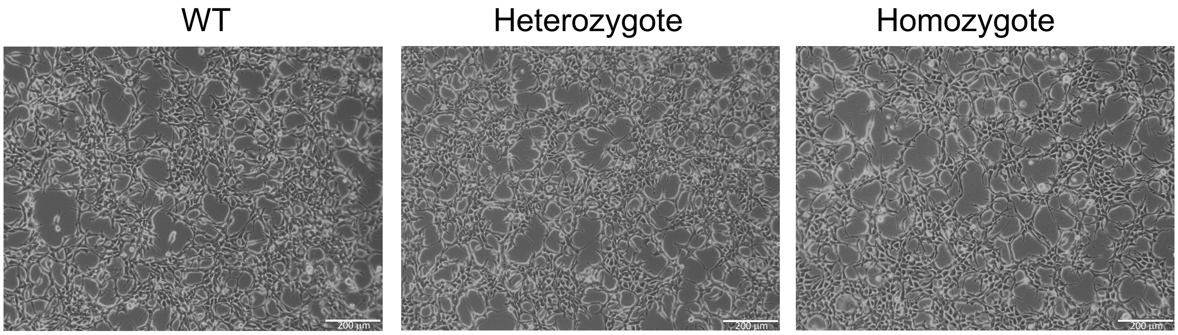

Supplementary Figure S14. Cells with PSMB6 T35A have normal morphology.

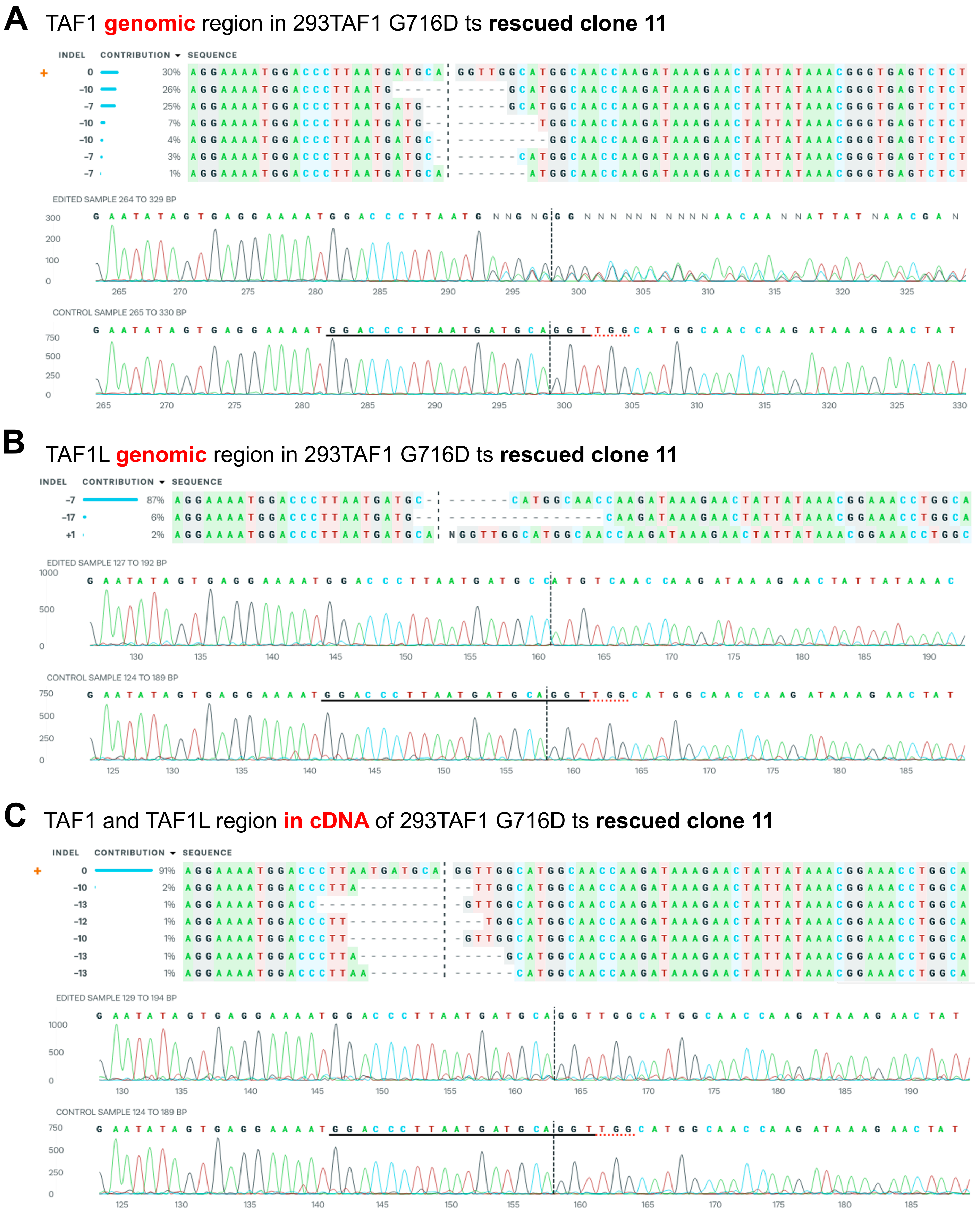

Supplementary Figure S15. Only wt TAF1 is expressed in rescued ts cells. A rescued clone of HEK293 TAF1ts clone 11 was analyzed by sequencing the genomic loci of TAF1 (A) and TAF1L (B), as also shown in Sup. Figures S4,5. Sequences were analyzed using Synthego ICE analysis tool. This sequencing shows the rescued TAF1 ts allele restored to wt, as well as the alleles with -7 and -10 deletions. The TAF1L locus has -7 and -17 deletions. Sequencing of the cDNA (C) showed that only the wt allele was detected, meaning that the deleted alleles were absent from the mRNA, likely due to nonsense-mediated decay.

Supplementary Figure S16: Raw images of blots

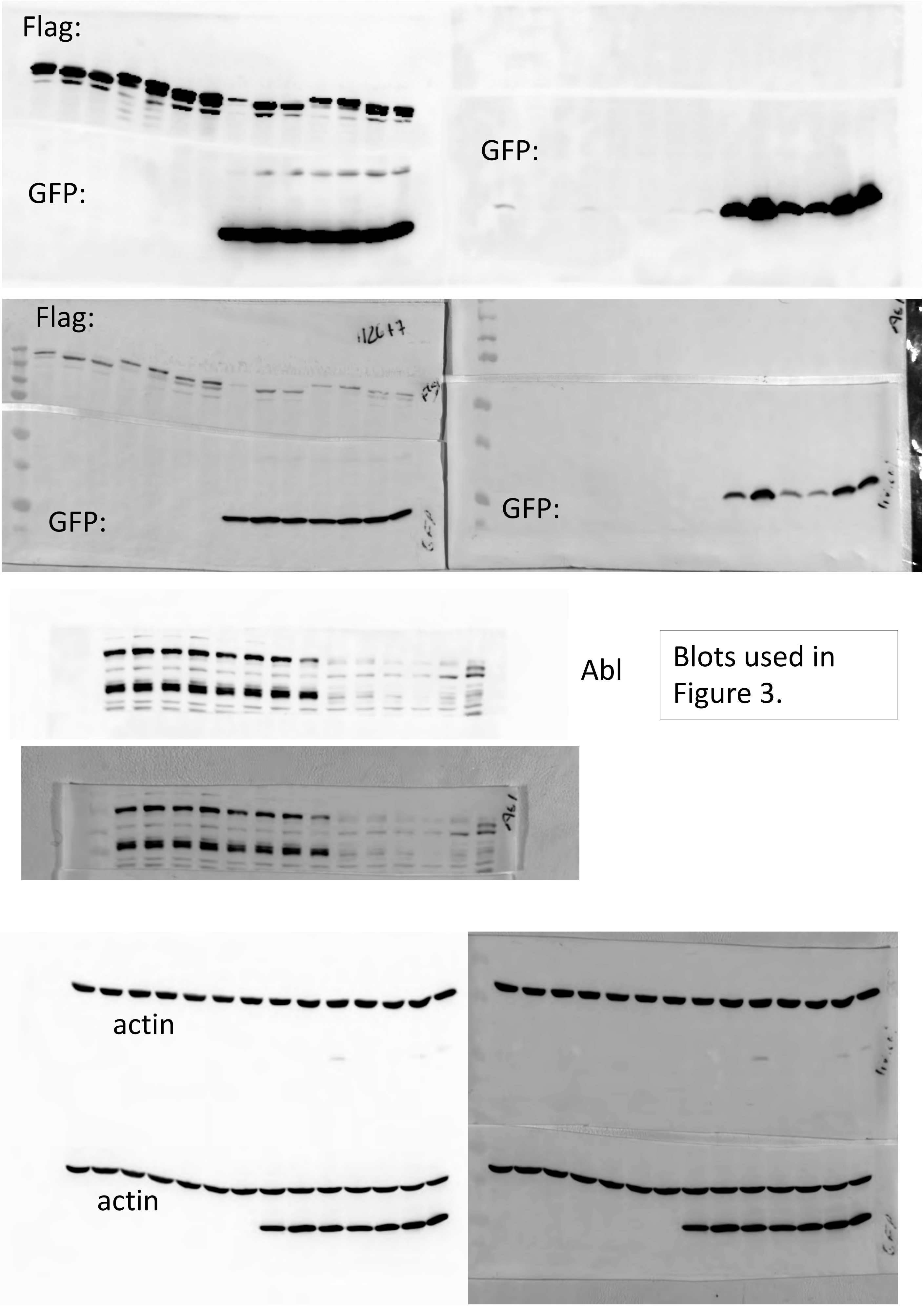

Blots used in Figure 4

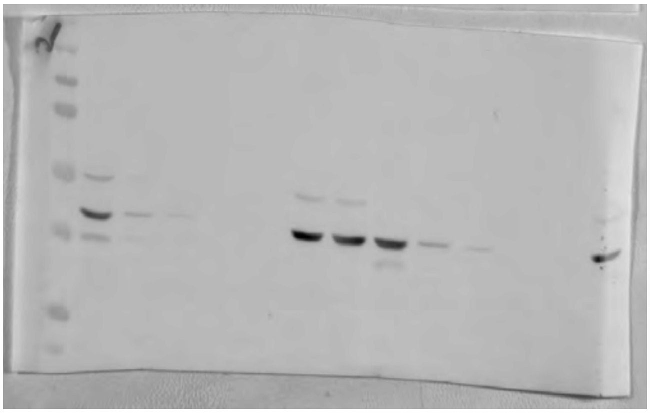

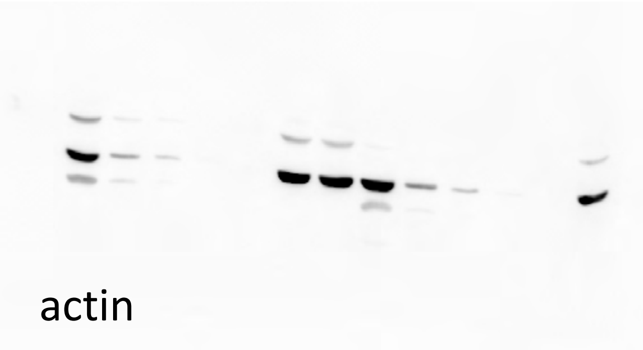

actin

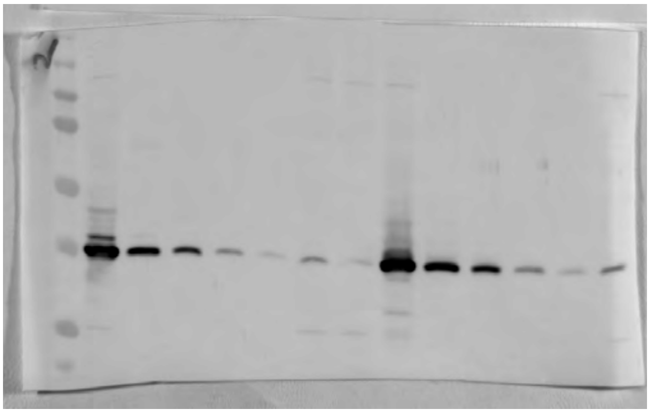

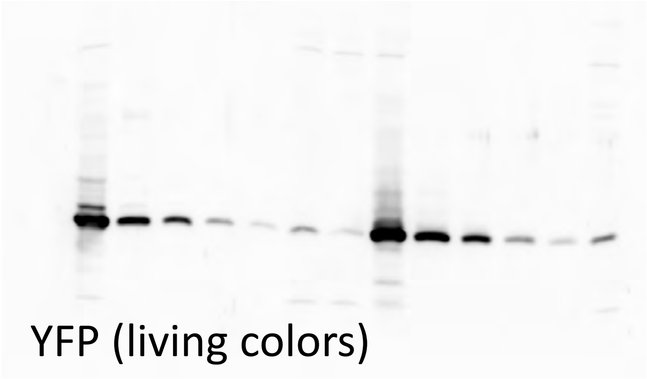

YFP (living colors)

Flag:

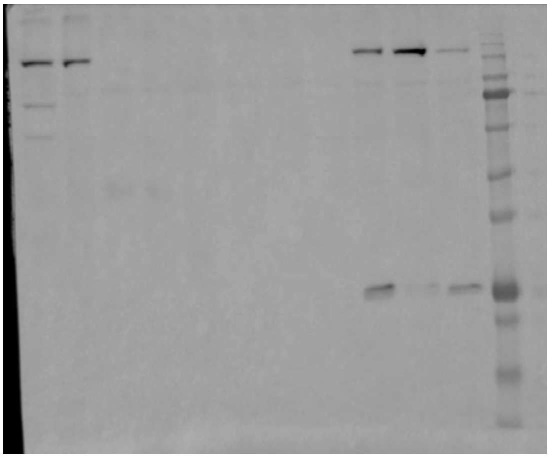

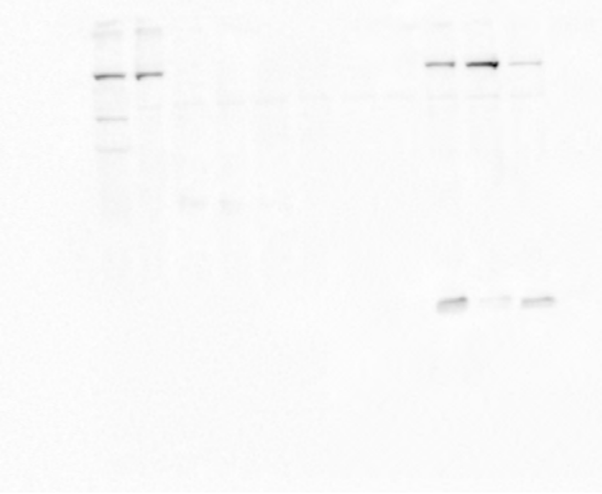

Actin:

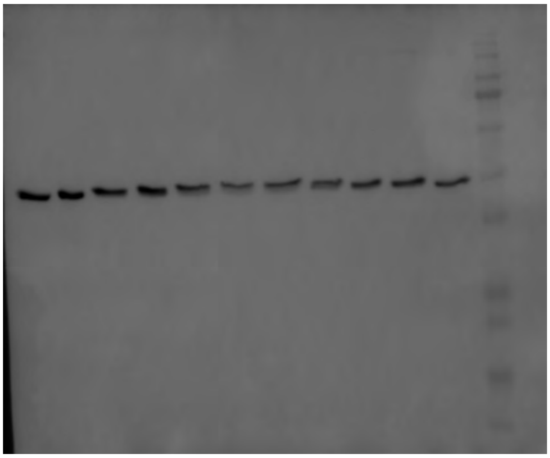

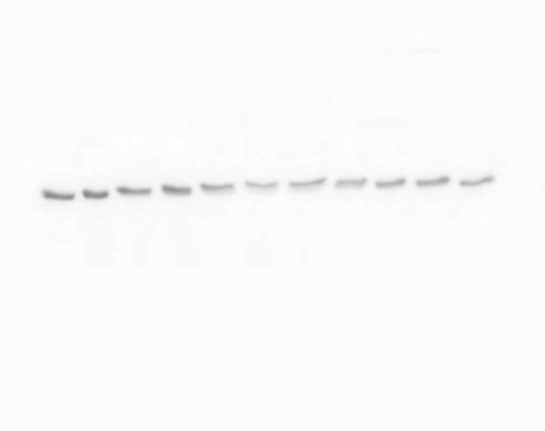

Blots used in Figure 5.

Living colors (YFP)

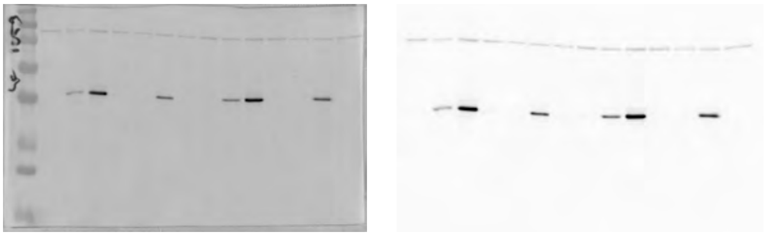

Actin

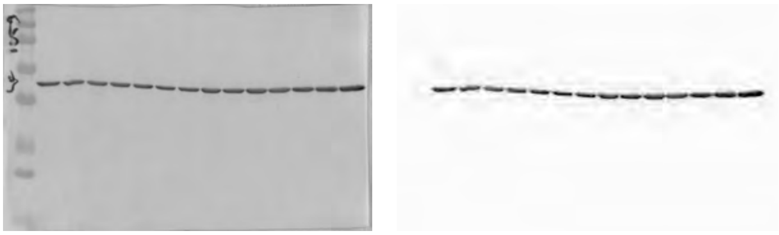

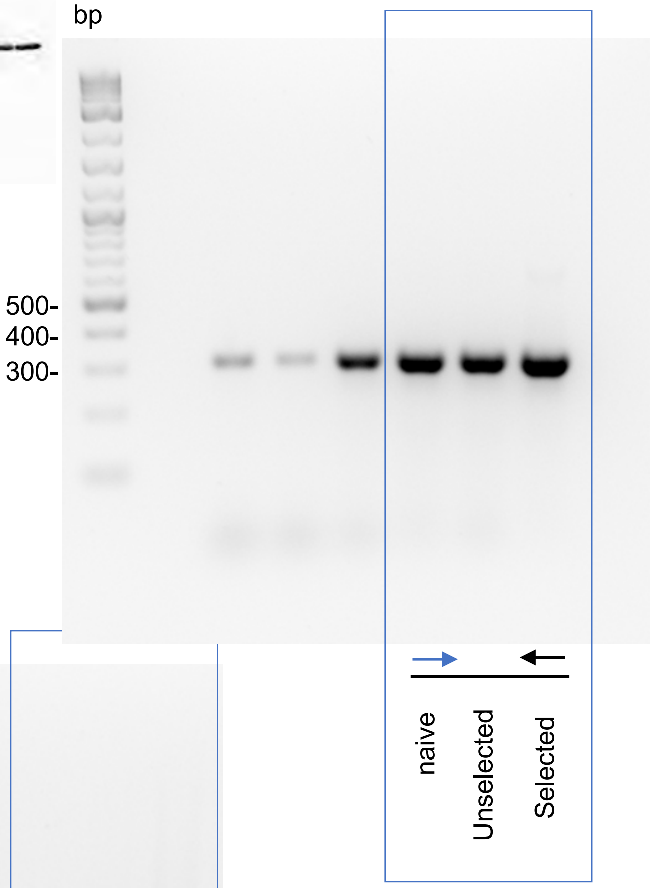

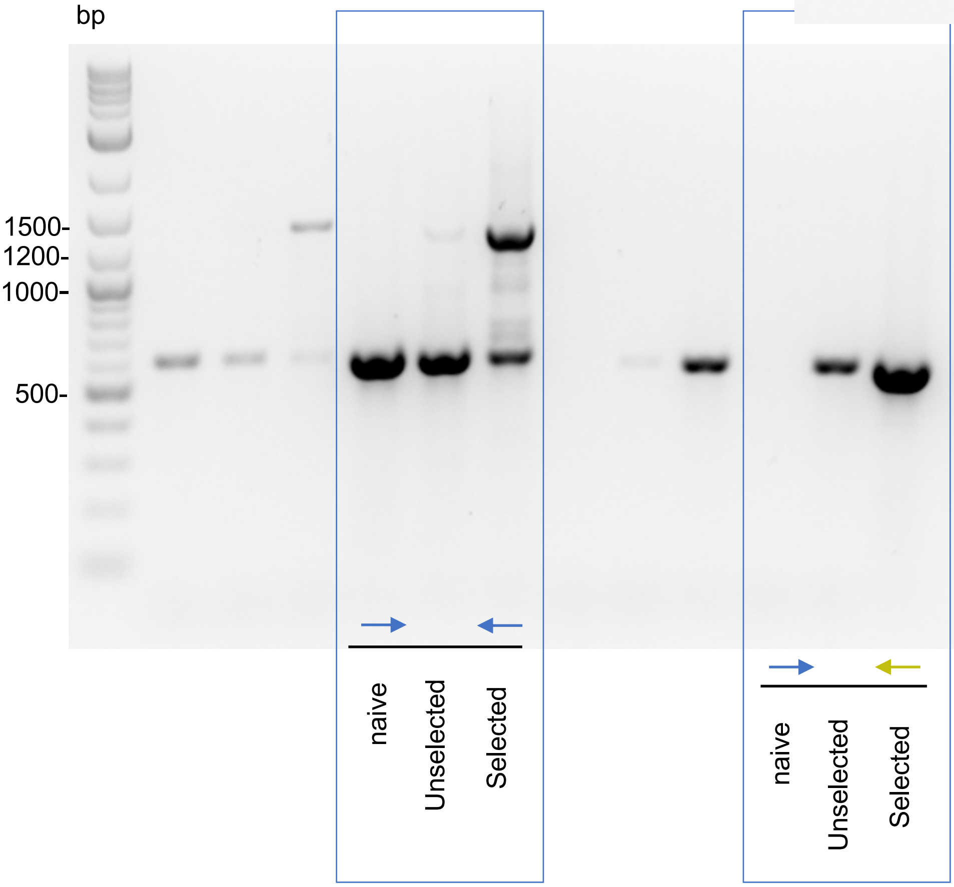

Supplement: Supplementary file 1 [file ijms-22-03741-s001.pdf]
